# Neurobartonelloses: emerging from obscurity!

**DOI:** 10.1186/s13071-024-06491-3

**Published:** 2024-10-05

**Authors:** Janice C. Bush, Cynthia Robveille, Ricardo G. Maggi, Edward B. Breitschwerdt

**Affiliations:** Intracellular Pathogens Research Laboratory, Comparative Medicine Institute, College of Veterinary Medicine, North Carolina State University, Raleigh, NC USA

**Keywords:** *Bartonella*, Bartonellosis, Neurological, Neuropsychiatric, Central neuropathy, Peripheral neuropathy, Cat scratch disease, Vector-borne, One Health

## Abstract

**Background:**

*Bartonella* species are fastidious, intracellular bacteria responsible for an expanding array of human pathologies. Most are considered to be transmitted by direct inoculation with infected bodily fluids from a mammalian reservoir species or vector-transmitted through a variety of arthropod species and their excrement. However, there are mounting reports of infection in the absence of documented animal or vector contact. A variety of *Bartonella* species have been documented in conditions affecting both the peripheral and central nervous systems. More common conditions, including neuroretinitis, are often associated with *Bartonella henselae*. However, *Bartonella quintana*, the agent of trench fever, as well as emerging pathogens related to rodent reservoir species, *B. grahamii* and *B. elizabethae*, have also been documented. Encephalitis and encephalopathy, also most often associated with *B. henselae*, have been reported with *B. quintana*, *B. washoensis* (ground squirrels) and *B. vinsonii* subsp. *vinsonii* (voles) infections. *Bartonella* infections have also been associated with peripheral neuropathies, such as cranial nerve paresis and neuropathic pain, including infection with less commonly encountered species such as *Bartonella koehlerae*. Recently, molecular diagnostic testing revealed that DNA from *Bartonella* spp. was found to be more prevalent in blood of patients with neuropsychiatric disorders such as schizophrenia and psychoses compared to healthy controls.

**Methods:**

A systematic literature search was conducted on PubMed, Google Scholar and Web of Science. Search terms included *Bartonella* and specific neurological conditions and focused on peer-reviewed case reports published after 2012 pursuant to a prior review, with limited exceptions for conditions not previously covered. Published diagnostic testing, serology, molecular testing or pathology, were necessary for inclusion, except for one case which had clinical and epidemiological evidence consistent with diagnosis along with follow-up.

**Results:**

Neurobartonelloses included neuralgic amyotrophy, complex regional pain syndrome, chronic inflammatory demyelinating polyneuropathy, cranial nerve paralysis, Guillain-Barré syndrome, peripheral vasculitic polyneuropathy, acute transverse myelopathy, neuroretinitis, encephalitis/encephalopathy, cerebral vasculitis/aneurysm and neuropsychiatric conditions.

**Conclusions:**

The breadth of reported symptoms and clinical syndromes associated with an increasing number of *Bartonella* species continues to expand. Increased clinical awareness of this important zoonotic pathogen is necessary to advance One Health among the medical and veterinary communities.

**Graphical Abstract:**

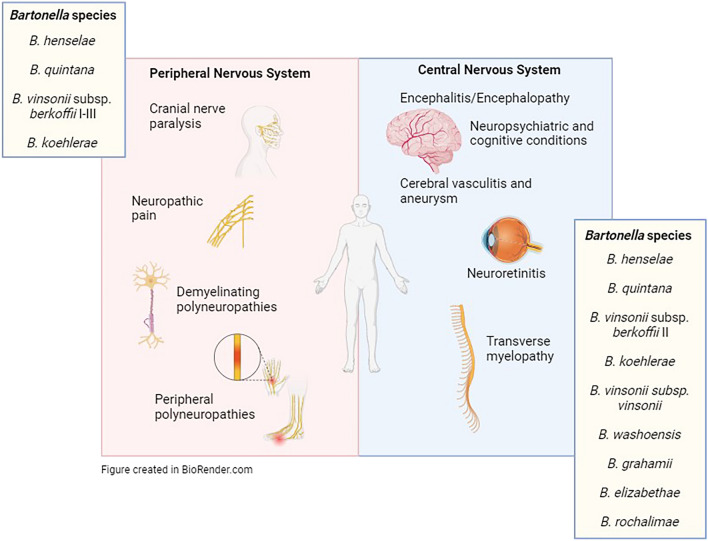

## Background

With the development of more sensitive and specific diagnostic testing modalities, *Bartonella* species are being increasingly recognized as important emerging human pathogens. In conjunction with an expansion in the basic microbiological, pathological and clinical understanding of these stealth bacteria, there is evolving evidence that this genus of bacteria is a more important contributor to neurological and neuropsychiatric illnesses than has been historically appreciated. Despite research progress to date, substantial gaps remain in our medical understanding of neurobartonelloses.

*Bartonella* species comprise an expanding genus of facultative intracellular Gram-negative bacteria that have coevolved in association with a large variety of mammalian species. These bacteria invade erythrocytes, vascular endothelium and other cell types and are capable of inducing long-lasting bacteremia [1–3]. Considered primarily vector-borne bacteria, arthropod transmission, most often among reservoir-adapted hosts, has been proven for some *Bartonella* species, whereas the vector remains unknown for other species [4, 5]. Direct inoculation of *Bartonella*-infected arthropod feces, or blood or bodily fluids from infected hosts, has also been reported via a scratch, bite or needle stick [1, 4–7]. Humans are considered the reservoir host for two species of *Bartonella*: *B. quintana* and *B. bacilliformis*. *Bartonella quintana*, the causative agent of trench fever, exists worldwide in association with its vector species, the human body louse (*Pediculus humanus humanus*), while *Bartonella bacilliformis*, the causative agent of Carrion’s disease, exists in a geographically isolated region in the Peruvian Andes [1]. Many other human pathogenic *Bartonella* species exist worldwide in mammalian reservoirs and vector species that environmentally overlap with human habitation, contributing to a high transmission risk to human beings [4, 8–12]. Since 1913, approximately 50 *Bartonella* species or candidates have been described in scientific publications [4]. In addition to humans, *Bartonella* species infect a wide range of wild and domestic animals, including bats, rodents, raccoons, foxes, deer, sheep, dogs and cats [4]. Periodically, *Bartonella* is transmitted from the reservoir host or its associated vector to a human being, who acts as an incidental host. In the context of emerging from obscurity, about 20 *Bartonella* species have been documented as zoonotic pathogens, of which nine have been reported in cases of neurological diseases. *Bartonella henselae*, the etiological agent of cat scratch disease (CSD), has been the most frequently implicated species. Named in 1931 by French physicians Robert Debre and George Semelaigne, “la maladie des griffe du chat” described suppurative adenitis in a 10-year-old boy also presenting with ipsilateral cat scratches [13]. Febrile illness and lymphadenopathy may precede development of symptoms in some cases of neurobartonellosis; nonetheless, there are several reported cases of neurological dysfunction following interaction with other animal species and cases where neither arthropod vector nor animal interaction is documented [2, 10].

Historically, the central nervous system (CNS) has been considered “immune privileged” because of several factors, including restricted pathogen access through the blood-brain barrier (BBB), a diminished immune response secondary to low MHC class I and II expression and a lack of a professional antigen-presenting cells [14, 15]. It is now known that the CNS routinely interacts with microbes and the systemic immune system and that these interactions, essential for brain homeostasis, are compromised during pathological conditions [14, 15]. Most recently, the effects of infectious organisms on chronic CNS disorders have been highlighted by the number of people suffering signs of cognitive impairment following coronavirus (SARS-CoV2) infection [16–18]. Data suggest that BBB dysfunction occurs in association with systemic SARS-CoV2 infection, with inflammation and vascular injury allowing for cytokine leakage into the CNS in the absence of the virus [18]. Of note, SARS-CoV2 infection has also unmasked or exacerbated preexisting bartonellosis, supporting the chronic, stealth nature of *Bartonella* infections [19, 20]. Evolving evidence supports a role for systemic inflammation due to *Bartonella* infection as well as pathogen presence within neurological tissues as causes of *Bartonella*-associated neurological dysfunction (BAND). In addition to erythrocytes and vascular endothelial cells, *B. henselae* infects a variety of other cell types, including macrophages, dendritic cells, mesenchymal cells and microglia, which may facilitate infection of nervous tissues [1, 2, 21–24].

This review will focus primarily on neurobartonelloses cases reported in the literature from 2012 through 2024, subsequent to the previous publication by Breitschwerdt, Sontakke and Hopkins that summarized case reports of neurological manifestations in immunocompetent patients secondary to *Bartonella* infection reported between 2005 and 2012. Selected cases published earlier or within that time period will be included to illustrate evolution in the medical understanding of neurobartonelloses and to expand on conditions not covered in the prior review. It will differ in scope from the previously published reviews, including those by Mazur-Melewska et al., which focused on the multi-system diseases associated with *Bartonella*; Canneti et al., which evaluated the frequency of neurological diseases in a retrospective study of *Bartonella henselae*-positive patients; Nawrocki et al., who focused on a variety of atypical cases of CSD; and Jurja et al., who gave an insightful overview of *Bartonella* and neuro-ophthalmological disorders and pathogenesis [25–28]. We will review cases of peripheral and central nervous system conditions related to infection with *Bartonella* species in conjunction with the mechanisms by which bacterial pathogenesis may relate to the development of neurological symptoms and pathology. Current diagnostic options and published treatment recommendations will be summarized. The purpose of this review is to further focus attention on the genus *Bartonella* as an underappreciated etiological agent that is being implicated in an increasingly wide spectrum of neurological presentations.

### Methods

This review is comprised of literature identified through electronic databases, including PubMed, Google Scholar and Web of Science, as well as the library system at the North Carolina State University. The review is organized by neuroanatomical location and primary clinical signs to include conditions afflicting the peripheral and central nervous systems. Inclusion criteria were peer-reviewed journal articles spanning the period of 2012 through July of 2024 as well as individual reports on specific neurological conditions from earlier time points not included in the previous review. Two cases documented in the previous review are included as examples of a novel *Bartonella* species causing neurological disease and to exemplify the range of neuropsychiatric symptoms possible in cases of *Bartonella* infection. Data on neurological disorders associated with *Bartonella* species consisted primarily of single- or multiple-patient case reports and a few review articles. Diagnostic methods including serology, molecular diagnostics or pathology with results were imperative for inclusion, although one case of facial nerve palsy was chosen to exemplify findings on abdominal imaging, which were consistent with disseminated cat scratch disease. Background information on neurological conditions was obtained from recent descriptive, pathogenesis or review articles specific to the condition. Search terms were used alone and in conjunction to decrease reporting bias, and searches were performed for both *Bartonella* and cat scratch disease in addition to the following terms: neurologic, neurologic disorders, neuropathy, peripheral neuropathy, peripheral nerve paresis, facial palsy, Bell’s palsy, neuropathic pain, peripheral polyneuropathy, Guillain-Barré syndrome, vascular neuropathy, spinal disease, spinal neuropathy, myelopathy, myelitis, acute transverse myelitis, meningitis, meningoencephalitis, central nervous system, encephalitis, encephalopathy, seizures, epilepsy, behavior, cognition, psychiatric, neuropsychiatric, immune-mediated, autoimmune, neuroophthalmological, optic nerve, neuroretinitis, cerebral vasculitis, cerebral aneurysm, infectious aneurysm, mycotic aneurysm, infectious neuropathy, pathogenesis, neuropathologic, pathological, coinfection and zoonoses. Additionally, human pathogenic *Bartonella* species, *B. henselae*, *B. quintana*, *B. vinsonii* subsp. *vinsonii*, *B. vinsonii* subsp*. berkhoffii*, *B. koehlerae*, *B. grahamii*, *B. washoensis* and *B. elizabethae*, were searched in association with neuropathies. All included references were read to completion by at least one author, and references within included manuscripts were also evaluated for additional case and background information. Most references were listed on PubMed.

### Clinical syndromes: peripheral neuropathies

Peripheral neuropathies are common, with an overall prevalence of 1–7% of the general population [29]. Etiology is variable, and up to 46% of cases are deemed idiopathic [29]. Sensory abnormalities, such as numbness, pain and loss of sensation, may be among the earliest symptoms and may progress to weakness and muscle atrophy. Symptoms related to autonomic dysfunction such as blurry vision, orthostatic hypotension and changes in urinary, gastric or bowel function may also occur in conjunction with the neuropathy [29]. Other reported symptoms include hyperalgesia, hyperesthesia, stabbing pain and allodynia (pain in response to a non-painful stimulus). In this section, we report on cases published between 2000 and 2024, as the previous review focused more on central neurological conditions.

#### Neuropathic pain

Neuralgic amyotrophy (NA), or brachial plexus neuritis, is a debilitating disease that classically presents as acute severe upper extremity pain followed by patchy muscle weakness and loss of sensation [30]. Overall incidence is presumed higher than the reported statistic of one per thousand individuals. Considered a multifocal, inflammatory condition, the etiology is thought to be autoimmune [30]. Although NA is often classified as idiopathic, it has been well described as a post-infection sequela related to multiple intracellular pathogens, including *Chlamydia pneumoniae*, *Borrelia burgdorferi*, group A *Streptococcus* and a variety of viral pathogens including SARS-CoV2 and Epstein-Barr virus (EBV) [30]. In cases of systemic infection with *B. henselae*, the mechanism underlying neuropathic symptomology is thought to be secondary to a CD8 + T cell immune response that leads to an autoimmune response, resulting in nerve damage. Due to the lack of patient response to antibiotic therapy, immune aspects of this disorder rather than direct bacterial effects have been hypothesized. It is thought that a disruption in the blood-nerve barrier allows for focal inflammation, with pain and nerve damage occurring secondary to inflammatory mediators and ischemia [30]. In the four cases presented in the literature (Table 1), unilateral and bilateral disease is reported, and three patients experienced a prior febrile illness and localized lymphadenopathy weeks to months prior to the onset of NA [31, 32]. Electroneuromyographic (EMG) features of denervation are consistent with NA, and patients often suffer significant loss of motor and sensory function lasting weeks to years. Whether due to antimicrobial or immunomodulatory effects, intravenous immunoglobulin (IVIG) may be therapeutically beneficial, allowing for near complete recovery in one documented case of NA neurobartonellosis [31, 32].

**Table 1:** Neuropathic pain disorders related to infection with *Bartonella* species

Peripheral neuropathies: neuropathic pain disorders								
Diagnosis	Patient age/sex	Initial symptoms/ clinical findings	Elapsed time to neurological symptoms	Key diagnostic results	Bartonella serology/PCR	Treatment/ duration	Outcome	Refs.
Neuralgic Amyotrophy (NA)	48/M	Fever, hepatic and splenic granulomas	6 weeks Left shoulder pain and weakness	Elevated CRP Leukocytosis MRI/CSF NSF	*Bh* IgM > 1:16 PCR neg. (liver)	Doxycycline + rifampin × 7 days	Resolution of fever and granulomas, recovery over several months	[31]
	53/M	Recurrent cutaneous abscesses, suppurative lymphadenitis and arthralgia	12 months Digital pain, right arm weakness	MRI/CSF NSF	*Bh* IgM > 1:16 PCR + (abscess)	Not reported	Resolution after 18 months	[31]
	46/M	Fever, enlarged inguinal LN	1 week Bilateral shoulder and arm pain and weakness	Elevated CRP MRI/CSF NSF	*Bh* IgM > 1:16 PCR + (LN)	Doxycycline + rifampin unknown duration	Resolution of inflammatory parameters, persistent pain and paresis for 18 months until lost to follow-up	[31]
	50/M	Abscess at site of scratch of unknown origin, fever, lymphadenopathy	2–4 days Electric shock sensations, right arm/hand pain/paresthesia, right thigh numbness	Elevated CRP Leukocytosis CSF specific oligoclonal bands Extensive infectious and autoimmune NSF_1_	*Bh* IgG > 1:1024 PCR neg. (CSF, LN) *Bh* IgG > 1:4096 after starting azithromycin	Amoxi-clavulanate	Initially prescribed for abscess	[32]
						Azithromycin	Switched to after onset of NA signs	
						Prednisolone	No change to neuro deficits	
						IVIG 6 cycles	Resolution with minimal residual signs	
Complex regional pain syndrome type I (CRPS)	28/F	Spider bite on right arm	1 week Ankle arthralgia and edema, persistent leg pain. Progression to erythema, cyanosis and burning sensation in bilateral feet, bilateral legs hyperalgesia	Extensive infectious and autoimmune panels NSF_2-4_	*Bvb*I IgG > 1:256, repeat > 1:1024 *Bvb*II IgG > 1:64, repeat > 1:128 *Bk* IgG > 1:256 *Bk* PCR + (blood, serum and culture-enriched blood)	Ibuprofen, prednisone, aspirin	Pain unresponsive	[35]
						Tramadol + gabapentin	Decreased pain and sensory signs	
						+ Nortriptyline	Slow improvement over weeks	
						Rifampin + azithromycin × 12 weeks	Progressive improvement and regain of function over 3 months	

Additional laboratory diagnostics denoted by subscripted numbers in diagnostic testing column. Sequential or repeated tests are identified by sequential numbers

^1^Negative for *Toxoplasma gondii*, *Treponema pallidum*, *Borrelia burgdorferi*, HIV, Hepatitis B, C, E. Vasculitis and ganglioside antibodies negative

^2^No abnormalities on CBC, serum biochemistry panel, urinalysis, ESR, CRP, ANA, RA, antistreptolysin

^3^Repeat CRP, ANA, antistreptolysin within normal limits

^4^HIV, syphilis, Lyme (*Borrelia burgdorferi*) negative

*PCR* polymerase chain reaction, *CRP-C* reactive protein, *MRI* magnetic resolution imaging, *CSF* cerebrospinal fluid, *NSF* no significant findings, *Bh* *Bartonella henselae*, *BvbI* *Bartonella vinsonii* subspecies *berkhoffii* type I, *BvbII* *Bartonella vinsonii* subspecies *berkhoffii* type II, *Bk* *Bartonella koehlerae*, *HIV *human immunodeficiency virus, *CBC* complete blood count, *ESR* erythrocyte sedimentation rate, *ANA* antinuclear antibodies, *RA* rheumatoid factor (rheumatoid arthritis)

Another neuropathic pain disorder, complex regional pain syndrome (CRPS), is typified by spontaneous onset of pain, numbness or burning, altered skin temperature or color, and varying levels of loss of motor function, most commonly affecting a distal limb, presenting days to weeks following an injury [33]. Pain is disproportionate to the inciting injury; the latter cannot be determined in some cases [33]. Most patients present with unilateral limb pain, but there are cases where pain occurs in multiple limbs or other body parts [33]. Vascular changes to the skin, including skin surface temperature changes, skin discoloration and swelling are common, and symptoms can spread to previously unaffected areas of the body [34]. Two primary classifications of CRPS exist based on specific nerve damage: Type I, the more common clinical presentation, occurs following trauma or an illness unaffiliated with a specific nerve. Type II CRPS occurs after injury to a specific nerve [33]. Proposed pathophysiological mechanisms that cause CRPS are thought to be multifactorial and include inflammation, autonomic nervous system alteration in which there are shifts in vascular and neuronal sensitivity to neurotransmitters, and CNS sensitization secondary to chronic peripheral nociceptor stimulation [34]. The role of the CNS in propagating CRPS appears linked to spinal cord glial activation that potentiates cytokine messaging and sensory transmission of pain stimuli [33, 34]. Type I CRPS has been documented in one case of *Bartonella* infection, without a defined preceding injury, in which a patient developed ankle pain that progressed to intermittent edema, erythema and cyanosis of both feet following a febrile episode that occurred after a spider bite on an upper extremity [35]. The patient, a veterinarian in Raleigh, NC, was diagnosed with CRPS after autoimmune and neurodiagnostic testing did not elucidate any other etiology. *Bartonella koehlerae* DNA was amplified from the patient’s blood directly and following enrichment culture in *Bartonella*-*Alphaproteobacteria* growth media (BAPGM) [36], and she was *B. koehlerae* and *Bartonella vinsonii* subspecies *berkhoffii* type I and II seroreactive on sequential blood testing. Symptoms, which had caused debilitation to the point of requiring a wheelchair, were controlled with a combination of nortriptyline and gabapentin, but resolution was not attained until the patient completed a 12-week therapeutic antibiotic regimen consisting of rifampin and azithromycin [35].

#### Peripheral nerve paresis

Chronic inflammatory demyelinating polyneuropathy (CIPD) has been described in two patients in association with *Bartonella* infection [37, 38]. Considered an autoimmune condition, CIPD is predominantly diagnosed in older male individuals and classically presents with an insidious onset, followed by progressive signs of relapsing and recurring symmetric peripheral muscle weakness, sensory alterations and paresthesia [39]. Loss of reflexes, neuropathic pain, autonomic dysfunction and cranial nerve abnormalities can coexist because of segmental demyelination related to inflammatory cell infiltration within the nervous system [39]. Infection or immunization has also been associated with the development of CIPD, and this population tends to be of younger age [40]. There is a case report of a 3-year-old male subject who developed symmetric distal muscular weakness and numbness with loss of deep tendon reflexes 6 weeks after being treated for lymphadenitis secondary to *B. henselae* infection (Table 2) [37]. Diagnosis of CIPD was based on clinical signs in combination with cerebrospinal fluid (CSF) findings of elevated monocytes, protein, oligoclonal banding and evidence of intrathecal IgG synthesis. Interestingly, serum IgG titers remained high following treatment with clarithromycin, which could indicate persistent infection. Functional recovery occurred over a period of 4 months with a tapering corticosteroid dose [37]. The second case involved a family that suffered from woodlouse hunter spider bites in their home following a flood and subsequent infestation of woodlice [38]. Spiders had been visualized on both sons and in their bedding. Suspected spider bites on the youngest son were documented by the child’s pediatrician when he was 5 months of age, and signs of muscle weakness and pain developed in toddlerhood. After an initial diagnosis of Guillain-Barré syndrome (GBS), this patient was subsequently diagnosed with CIPD following episodic relapsing weakness and evidence of demyelination on EMG (Table 2). This patient displayed rising titers during serial testing to several *Bartonella* species, which diminished after antibiotic therapy (refer to Table 2). His older brother also experienced symptoms following spider bites, including disruptive sleep, and both boys developed anxiety, irritability and panic attacks that could not be attributed to another somatic disease. The older son developed cervical lymphadenopathy about a year after experiencing the spider bites, and his mother, although she could not confirm being bitten by a spider, experienced headaches, eye pain, weakness and loss of sensation in her extremities along with joint pain, fatigue and neurocognitive signs of memory loss, insomnia and disorientation in the months pursuant to the presence of spiders in the dwelling. Interestingly, all three family members tested positive by immunofluorescent antibody (IFA serology) to *B. henselae* San Antonio 2 (SA2) and *B. vinsonii* subsp*. berkhoffii* type II, although at a lower titer than the youngest son. There was no history of cat interaction, although the family dog had a history of fleas. The dog’s blood was screened during the same time interval as the mother and found to be serologically negative to all tested *Bartonella* species [38]. Thirteen spiders and four woodlice were collected from the home for *Bartonella* screening. Compellingly, *B. henselae* SA2 DNA was genetically sequenced from two spiders and one woodlouse and *B. vinsonii* subsp. *berkhoffii* sequenced from a third spider [38]. Although vector competency was not confirmed for these species, the findings bear consideration due to the temporal association of spider bites and the development of bartonellosis [38].

**Table 2:** Peripheral nerve paresis associated with *Bartonella* species infection

Peripheral neuropathies: peripheral nerve paresis								
Diagnosis	Patient age/sex	Initial symptoms/clinical findings	Elapsed time to neurological symptoms	Key diagnostic results	Bartonella serology/PCR	Treatment/ duration	Outcome	Refs.
Chronic inflammatory demyelinating polyneuropathy (CIPD)	3/M	Lymphadenitis	6 weeks Symmetric distal muscle weakness, sensory ataxia, ↓ to absent deep tendon reflexes	Infectious and autoimmune panels NSF_1_ CSF mild ↑ protein, oligoclonal bands and slight intrathecal IgG synthesis. Decreased motor neuron conduction	Bh IgG > 1:850 IgM > 1:250 Serum PCR neg CSF serology and PCR neg	Clarithromycin + prednisone taper over 4 months	Recovery of motor and sensory function, reflexes, repeat titers IgM < 1:250, no change to IgG. Nerve conduction normalized at 1 year	[37]
	2/M	Previous history of woodlouse hunter spider bites, intermittent rashes and sinusitis	2 years Ataxia, leg pain, dizziness, visual floaters and constipation	CSF: ↑ protein. MRI: enhancement of ventral nerve roots/pia from 11th thoracic vertebrae to sacrum	Not initially obtained	IVIG × 4 d. for suspected GBS	Rapid improvement then relapsing muscle weakness 2 months later	[38]
			Weakness and pain in legs, tingling around mouth	EMG: chronic sensory motor demyelinating polyneuropathy	*Bh* IgM/IgG not detected at 1:16	IVIG + prednisone + gabapentin × 4 w Azithromycin × 10 d	Antibiotic helpful, relapsing muscle weakness 1 year later	
			Weakness in legs	None noted	Titer 1: + *Bh, Bk*, Titer 2: + *Bvb* I-III, *BhSA2*/*H1* and *Bk* Titer 3: all decreasing	IVIG Azithromycin × 30 d	Improvement on antibiotics and IVIG	
						Taper of IVIG Clarithromycin + rifampin × 6 months	Full recovery with minimal stiffness in legs after 2 months	
Cranial nerve paralysis	29/F	Fever, night sweats, headache, left parotid enlargement and facial weakness	5 weeks OS ptosis, mydriasis and enlarged occipital LN	MRI: regional lymphadenopathy Histopathology: granulomatous inflammation	*Bartonella* sp. positive serology (not specified)	None	Complete recovery over 4 months	[51]
	28/M	Headache, fever, fatigue and myalgia × 3d, followed by right preauricular LN swelling 1 week later	16 days Blurred vision, right eyelid weakness	CBC, EBV, CMV NSF	No initial Bartonella diagnostics	Prednisone	Improvement with relapse upon treatment cessation	[49]
			1 week later Recurrent ocular, facial nerve symptoms, fever, chills and myalgia. New ↑ cervical LNs	CBC mild ↑WBC, viral and STI screening NSF_3_ CT: cortical necrosis right preauricular LN and parotid sialadentis	Bh IgG > 1:640 IgM neg Bq neg	Azithromycin × 5d	Resolution 2 weeks after treatment	
	7/M	Transient fever, cat scratch left cheek	1 month Left facial palsy with recurrent fever and cervical lymphadenopathy	WBC and CRP normal	No initial Bartonella diagnostics	IV Acyclovir + prednisolone	Fever initially responded then relapsed	[47]
				Repeat WBC ↑, CRP normal CSF normal		Flomoxef + azithromycin	No clinical improvement, WBC returns to normal	
				Ultrasound/MRI cervical/parotid swelling + facial nerve compression	Bh IgG > 1:1024 IgM neg	Minocycline + ceftriaxone	Improvement	
						Amoxicillin + pred	Resolution over 6 months	
	5/F	19-day history of fever, headache, fatigue, weight loss. Strep. pharyngitis	21 days Left sided facial palsy	↑CRP, ESR. Blood culture and cranium CT NSF. Infectious disease testing NSF_4_.Ultrasound: hypoechoic splenic and hepatic foci. Brain MRI NSF	Suspected disseminated CSD, no specific Bartonella diagnostics	Azithromycin + Rifampin × 14d	Resolution of all clinical signs	[48]

Subscripted numbers indicate additional laboratory findings associated with individual patients

^1^Negative for adenovirus, respiratory syncytial virus, coronavirus, influenza, parainfluenza, Sendai virus, mumps, measles, herpes simplex, varicella zoster, *Mycoplasma pneumoniae*, *Chlamydia psittici, Coxiella burnetti, Mycobacterium tuberculosis*, atypical *Mycobacterium*. Enolase, myelin basic protein autoantibodies, glucose and lactate normal. ANA, ANCA and complement 3, 4 normal

^2^Blood draws for titers were obtained every other day for 3 samples. Titer 1: Bh SA2 and Bh H1 1:256, Bk 1:128. Titer 2: Bvb I 1:64, Bvb II 1:1024, Bvb III 1:128, Bh SA2 1:512, Bh H1 1:512, Bk 1:2048. Titer 3: Bvb I 1:32, Bvb II 1: 512, Bvb III 1: 128, Bh SA2 1:256, Bh H1 1:256, Bk 1:512. All negative by 2 months follow-up

^3^Gonorrhea, chlamydia PCR, HIV antigen/antibody, plasma reagin, hepatitis B and C, Epstein-Barr virus IgM, cytomegalovirus IgG, LDH all negative/normal

^4^Rickettsia, Lyme (*Borrelia*), West Nile virus, varicella, mumps, Epstein-Barr virus, cytomegalovirus and herpes simplex virus 1 and 2

*NSF* no significant findings, *CSF* cerebrospinal fluid, *Bh* *Bartonella henselae,* *PCR* polymerase chain reaction, *LN* lymph node OS-left eye, *MRI* magnetic resonance imaging, *CBC* complete blood count, *EBV* Epstein-Barr virus, *CMV* cytomegalovirus, *WBC* white blood cells, *STI* sexually transmitted infections, *CT* computerized tomography, *Bq* *Bartonella quintana,* *CRP* C reactive protein, *IV* intravenous, *ESR* erythrocyte sedimentation rate, *CSD* cat scratch disease. *ANA* antinuclear antibody, *ANCA* anti-neutrophil cytoplasmic antibody, *LDH* lactate dehydrogenase

Facial nerve paresis, or Bell’s palsy, is the most commonly reported cranial nerve paresis. Up to 75% of adult cases are considered idiopathic, with traumatic causes making up an additional 10–23% [41]. Infection is cited in comparatively few cases, but it may be more prevalent when facial nerve paresis is combined with other systemic symptoms [41]. General symptoms include abrupt onset of unilateral facial muscle weakness resulting in incomplete closure of the eyelid, lack of forehead wrinkling and labial droop. The incidence varies with age, being less prevalent in children < 15 years old. Etiology in children, however, is more likely to be infectious, accounting for up to 36% of cases, as opposed to 3.1% of cases in adults on average [42]. In terms of infectious pathogenesis, acute otitis media secondary to bacterial infection is cited as the most common cause of facial palsy in children, although *B. burgdorferi*, the spirochetal organism responsible for Lyme disease, is deemed responsible for up to 50% of facial palsy in children living in Lyme-endemic regions. This may relate to the location of *B. burgdorferi* inoculation in this population, in which tick bites on the head or neck are more common [43, 44]. As *Bartonella* and *Borrelia* can coinfect and the organisms have been documented in the same arthropod vectors, *Bartonella* may go untreated if clinical symptoms are assigned solely to Lyme disease [43–46]. In cases of neurobartonelloses, facial palsy is commonly associated with other systemic symptoms (Table 2). Of the nine *Bartonella* cases documented in the literature since 2005, four cases are reported in children/adolescents < 18 years old [47–50]. Fever was common in all cases. Concomitant headaches and weight loss, granulomatous lymphadenopathy and parotid swelling were noted, respectively, in three patients [47–49]. The fourth pediatric patient, who was co-infected with herpes simplex virus (HSV), presented with ocular pain and loss of vision [50]. In the adult cases, fever was reported in three of the five patients, and regional parotid swelling was commonly noted [49, 51–53]. Two patients presenting with facial palsy also developed Parinaud’s oculoglandular syndrome (discussed further in the section on Neuroretinitis), illuminating this pathogen’s ability to incite multi-focal pathology. The first of these patients, a 28-year-old man, developed fever and malaise followed by upper eyelid weakness ipsilateral to conjunctival signs, with compression of the facial nerve secondary to enlarged pre-auricular lymph nodes and parotid sialadenitis. He reported being scratched by kittens adopted 2 months prior to the onset of clinical signs, although the localized ocular symptomology may suggest inoculation through the conjunctiva. Serology revealed *B. henselae* IgG at 1:640, consistent with active or recent infection [52]. The second patient, a previously healthy 47-year-old woman, presented with left-sided palsy of the abducens nerve and dipoplia. Her condition progressed over a 4-day period to visual loss secondary to neuroretinitis. CSF revealed evidence of aseptic meningitis, and upon further questioning, the patient recalled a “flu-like illness” and cat contact 2 weeks prior to the onset of her clinical signs.

 Subsequent *Bartonella*serology was determined to be positive [53]. Table 2 includes four cases of peripheral nerve palsy chosen to illustrate variation in clinical presentation, laboratory and imaging parameters, and patient outcome.

### Peripheral polyneuropathies

#### Guillain-Barré Syndrome

Guillain-Barré syndrome (GBS) is a polyradiculoneuropathy that arises from autoimmune damage to peripheral nerves [54]. Two primary subtypes of GBS exist. The more common subtype, acute inflammatory demyelinating polyneuropathy (AIDP), involves immune-mediated damage of the peripheral nerve myelin sheath, whereas in the second subtype, acute motor axonal neuropathy (AMAN), the immune injury involves the axolemma (axonal membranes) [55]. The most common presentation is acute onset of ascending weakness and hyporeflexia, typically arising from the lower limb(s), which may be preceded by paresthesia and/or pain [56]. Although a genetic predisposition is suspected, a seasonal variation associated with infectious disease outbreaks also exists [58]. Patients commonly report a previous illness, often accompanied by respiratory or gastrointestinal symptoms. Outbreaks of GBS have been associated with multiple pathogens, including Zika virus, SARS-CoV2 and *Campylobacter jejuni* [56, 57]. Incidence increases with age, and the condition is more common in females. In a case report by Massei et al., a 10-year-old female patient presented with loss of mobility in her lower limbs, with a 1-day history of vomiting and fever 4 days prior to the onset of neurological signs [58]. Weakness, myalgia and loss of deep tendon reflexes in the lower extremities were noted, along with pelvic and truncal weakness. An extensive diagnostic workup (Table 3) was normal aside from reduced motor nerve conduction velocity and amplitude, consistent with axonal damage. This highlights a neurobartonellosis case with no classical signs of CSD such as fever and lymphadenopathy. Screening for *Bartonella* was elected because of the patient’s history of living in a rural setting and having previous kitten interactions. Elevated titers to *B. henselae* (IgG 1:1024 and IgM +) were detected on blood serology, while CSF was negative via polymerase chain reaction (PCR). The patient demonstrated rapid response to IVIG and was discharged with no lingering neurological deficits.

**Table 3:** Polyneuropathic conditions associated with *Bartonella* species infection

Peripheral neuropathies: polyneuropathic conditions								
Diagnosis	Patient age/sex	Initial symptoms/ clinical findings	Elapsed time to neurological symptoms	Key diagnostic results	Bartonella serology/PCR	Treatment/duration	Outcome	Refs.
Guillain-Barré syndrome	10/F	Fever and emesis	4 days Difficulty walking and myalgia progressed to generalized weakness, loss of deep tendon reflexes in lower limbs, and pelvic and truncal weakness over pursuant 3 days	CBC: mild neutrophilic leukocytosis and thrombocytosis CSF and AUS NSF Infectious/immune disease panel NSF_1_ Reduced motor n. conduction velocity/amplitude	*Bh* IgG 1:1024 IgM + CSF PCR neg	IVIG × 5 days	Rapid resolution of neurological signs with no remaining deficits, *Bh* IgG titer dropped, and specific IgM disappeared	[58]
Vasculitic polyneuropathy	40/M	Acute onset arthralgia, swelling and pain in hands, Raynaud’s syndrome, swelling and ulceration of digit 2 right foot, *livedo reticularis* bilateral lower limbs	2 years Progressive asymmetric polyneuropathy, hyperesthesia and hyperalgesia of right leg, lower left leg, fingertips	Reduced nerve conduction velocity in legs, neurogenic changes to skeletal muscle Biopsy: axonal neuropathy with inflammation ↑ACE and IgA. ↓ C3 CSF NSF *Borrelia burgdorferi* and *Treponema pallidum* neg	No testing	High-dose corticosteroids	No change to neurological signs	[64]
		5 years after initial onset: developed recurrent digital ulcer	Persistent neurological deficits	No diagnostics	No testing	None	No treatment sought	
		7 years after initial onset: recurrent digital ulcer, night sweats and weight loss	Persistent neurological deficits	↓ WBC and Fe, ↑ IgA and IgA immune complexes Extensive infectious/immune panel NSF_2_ Biopsy of ulcer: vascular proliferation	*Bh* IgG > 1024 *Bq* IgG 1:128	Erythromycin × 4 months	Improvement with residual pain in ulcerated toe, *livedo reticularis* and digital hypothermia	
					*Bh* titer dropped to 1:256	Doxycycline × 3 weeks	No recurrent ulceration with minimal residual polyneuropathy and Raynaud’s syndrome for following 4 years	

^1^Negative to EBV, CMV, *Campylobacter jejuni*, *Mycoplasma pneumoniae*, rubella, measles, mumps, influenza, parainfluenza, parvovirus B19, Coxsackie B virus. *Campylobacter jejuni* negative in fecal study

^2^CBC, lymphocyte subsets, standard chemistries, sedimentation rate, CRP, IgG, IgM, IgE, C3, C4, ACE, B-2 microglobulin, RF, C-ANCA, P-ANCA, C1q binding immune complexes, cryoglobulins, cold agglutinins, anti-cardiolipin, ANA, d-DNS, RNP, Ro, La, SCL 70, CENP-B and Jo all nsf. Hep-B, G, HIV, HHV-8 neg. *Borrelia burgdorferi* IgG ELISA positive with inconclusive WB

*CBC* complete blood count, *CSF* cerebrospinal fluid, *AUS* abdominal ultrasound, *NSF* no significant findings, *Bh* *Bartonella henselae*, *PCR* polymerase chain reaction, *IVIG* intravenous immunoglobulin, *ACE* angiotensin converting enzyme, *C3* complement 3, *WBC* white blood cells, *Fe* iron, *WB* Western blot

#### Peripheral vasculitic polyneuropathy

In recent years, the association between hematological and neurological diseases has become an increased area of investigation [59]. Peripheral vasculitic polyneuropathy is a multifactorial condition in which inflammation of the vasa nervorum, the complex of vessels which supplies nutrition to peripheral nerves from adjacent vasculature, results in thrombosis and secondary ischemic injury [60]. The vasculitis itself can be regional, affecting only a peripheral nerve, or more systemic, either primary, or secondary to autoimmune or infectious disease [59, 61]. Cases that present solely with peripheral neuropathy symptoms require a high index of suspicion to determine whether underlying vasculitic changes are driving the condition [59, 61]. In the case of infection, vascular damage can be indirect, from immune complex deposition or cell-mediated immune hypersensitivity, or arise directly because of pathogen influence. Some pathogens, including *Bartonella*, can cause damage by direct endothelial invasion [59, 61–63]. In the presented case, a patient infected by *B. henselae* endured relapsing cutaneous ulcerations on his feet and developed an asymmetrical peripheral neuropathy involving both legs and hands, with concomitant hyperesthesia and hyperalgesia over a period of a few years (Table 3) [64]. The patient, a 40-year-old male, initially presented with an acute onset of joint pain, digital swelling and pain, and livedo reticularis on his distal limbs. He also had symptoms of Raynaud’s phenomenon and a digital ulcer on the right foot. A muscle biopsy revealed axonal neuropathy, but treatment with corticosteroids was unsuccessful. Recurrent cutaneous ulcerations, night sweats and weight loss continued over several years. Further diagnostics, including an extensive investigation into immune-mediated and infectious diseases, were not conclusive. Due to the recurrent presentation of cutaneous ulcers, suspicion of bacillary angiomatosis was raised, and *Bartonella* serology was obtained (Table 3). Antibody titers to *B. henselae* were determined to be elevated, along with equivocal titers to *B. quintana*, and a tissue biopsy of the cutaneous lesion demonstrated subepithelial proliferation of small vessels [64]. The patient responded to doxycycline, and at follow-up, *Bartonella* titers had decreased, and the patient had only residual signs of neuropathy. This case is an extreme example of the chronic, insidious nature of *Bartonella* infections, underscoring the need for high clinical suspicion and collaborative efforts in determining an etiology.

### Central neuropathies

#### Spinal cord conditions

Depending on the location(s) of the offending lesion, patients with neuropathological conditions affecting the spinal cord can present with a spectrum of neurological symptoms, varying from exclusively motor to predominantly sensory abnormalities, or concurrent sensory and motor nerve deficits [65]. Due to overlap with common spinal conditions that manifest in sensory or motor signs, including degenerative myelopathy, intervertebral disc disease or nutritional deficiencies, diagnosis of primary infectious myelopathies is complicated. Symptoms can be secondary to spinal compression because of focal inflammation or to internal or external abscessation. Symptoms can also arise secondary to direct nervous tissue invasion by microbes, such as *Enterovirus* [66]. These para- and postinfectious presentations of spinal pathology can impact timely diagnosis [66, 67].

In cases of BAND, acute transverse myelitis (ATM) is the most commonly documented spinal condition, with or without overlapping GBS. Interestingly, these two conditions are being reported more frequently and are termed GBS/ATM overlap syndrome [68]. ATM is described as a rare disorder, in which spinal cord inflammation results in myelin damage. Although most commonly termed idiopathic, multiple infectious agents have been implicated, including *B. burgdorferi*, *Mycoplasma* and, most recently, SARS-CoV2, where post-infection and post-vaccination cases were seen with unexpectedly high frequency [68–71]. Most often, one or more focal inflammatory lesions traversing the thoracic spinal cord leads to bilateral motor weakness and other symptoms including autonomic dysfunction, sensory deficits and bladder or bowel dysfunction [70]. Partial involvement affecting only one side of the body can also occur [70]. ATM progresses rapidly, and by the time of peak neurological deficit, about half of patients are paraplegic. The disorder can be temporary (3–6 months) or permanent, with patient outcome roughly divided equally into one of three categories: complete resolution, moderate disability or severe, permanent disability [70]. Additionally, patients with ATM can develop other demyelinating disorders, such as multiple sclerosis (MS), at a later date [71]. Of interest, however, in the publication by Kim et al. (2023), biomarkers of neuronal and astroglial damage, though comparable in acute-onset patients with relapsing-remitting MS, do not remain elevated during remission. Lack of specific damage-associated biomarkers during remission suggests that MS is not prone to progression [72]. This finding underscores the need for more broad infectious disease testing in cohorts that do not achieve disease resolution. Four *Bartonella*-associated cases have been published since the prior neurobartonellosis review, all of whom were diagnosed with *B. henselae* (Table 4). Three patients reported a cat scratch or bite days to weeks prior to development of neurological symptoms, and the fourth patient had exposure to kittens and various arthropod vector species in her rural environment. In two of these ATM patients, a 10-year-old girl and a 62-year-old woman, the initial presenting signs of lower limb weakness, hyperalgesia and anuria led to a diagnosis of ATM, confirmed by the presence of spinal lesions on magnetic resonance imaging (MRI) [73, 74]. The older patient also had blood pressure exacerbations suggestive of autonomic dysfunction. Both had elevated CSF leukocyte counts and protein, and neither responded to ceftriaxone and vancomycin therapy. Once CSD was suspected because of history of cat interaction/bite wound, both patients demonstrated clinical improvement with doxycycline administration but still had significant residual pain, weakness and dysuria. Both patients were diagnosed with concurrent GBS when EMG documented diminished motor nerve conduction. Addition of IVIG resulted in full recovery in the older patient and clinical resolution with minor sensory deficits at a 4-month follow-up appointment in the younger patient (Table 4). A third patient, a 12-year-old boy with a historical cat bite injury, presented with inability to walk, anuria and fluctuating hypertension, similar to the 62-year-old female patient, who had also sustained a cat bite [74, 75]. In this case, however, a high-normal erythrocyte sedimentation rate (ESR), CSF findings of low protein and glucose, and evidence of a demyelinating polyneuropathy on EMG led to the initial diagnosis of GBS, after which IVIG therapy was instituted. Lack of improvement in clinical signs led to spinal imaging, which was consistent with ATM, and *Bartonella* antibody titers were elevated (Table 4). Addition of methylprednisolone, rifampin and doxycycline resulted in near complete recovery by the 4th day of therapy.  At a 1-month follow-up appointment, the boy had no residual neurological deficits aside from hypoactive deep tendon reflexes [75]. The fourth patient, a 46-year-old woman with a history of a cat scratch and axillary lymphadenopathy, developed a larger spectrum of clinical abnormalities [76]. Her ATM symptoms included acute onset of paresthesia, lower limb weakness and pain, and diagnosis was confirmed by MRI (Table 4). However, her additional symptoms included dysarthria, peripheral neuropathic features of dysphagia and facial paralysis, and central neuropathic signs including gaze-induced nystagmus and aphasia, suggesting central and peripheral nervous system involvement [76]. Cerebrospinal fluid monocyte, protein and lactate values were increased, and the CSF: serum albumin ratio supported blood-CSF barrier dysfunction. Of note, this patient had an extensive diagnostic workup for infectious and immune-mediated diseases prior to being assessed for neurobartonellosis (Table 4). *Bartonella henselae* serology was positive, as was PCR from a tissue biopsy obtained from the site of the cat scratch. *Bartonella* DNA was not PCR amplified from the CSF. The patient was treated with a 3-week course of doxycycline and a tapering dose of corticosteroids, and although she had improvement in her overall clinical status, she reported persistent fatigue, chronic headache and radicular nerve pain, and had gait deficits at a 6-month follow-up appointment [76].

**Table 4:** Acute transverse myelitis secondary to *Bartonella* infection

Central neuropathies: acute transverse myelitis							
Patient age/sex	Initial symptoms/ clinical findings	Elapsed time to neurological symptoms	Key diagnostic results	Bartonella serology/PCR	Treatment/ duration	Outcome	Refs.
10/F	Cervical lymphadenopathy, abdominal pain, vomiting and urinary retention	7 days Lower extremity weakness, lower back/neck pain, headache, burning sensation in bilateral carpi, stifles, tarsi and feet	CBC NSF. Mild ↑ ALT/AST MRI: Focal increased T2 weighted signal in brain and central longitudinal increased signal in spinal cord CSF: moderately ↑ lymphocytes, mildly ↑ protein, MBP ↑ Infectious disease/immune panel NSF_1_	*Bh* IgG 1:152, IgM 1:160	Ceftriaxone + vancomycin initiated Switched to rifampin + doxycycline × 14 days upon *Bh* results	Switched protocol on suspicion of CSD Rifampin d/c due to rising liver values Improved muscle strength, continued difficulty voiding and severe lower extremity pain	[73]
			Recheck CSF: ↑ lymphocytes Nerve conduction: patchy mixed demyelinating axonal motor and sensory neuropathy	Sequential rise in IgG, 1:256 to 1:512 Sequential drop in IgM 1:40 to 1:20	Added IVIG for suspected GBS	Resolution Minor residual sensory deficits at 4-month follow-up	
46/F	Cat scratch wound on finger followed by axillary lymphadenopathy 2 weeks later	4 weeks Acute onset paresthesia, lower limb weakness, dysphagia, dysarthria, facial paralysis, gaze-induced nystagmus and aphasia	↑ WBC, CRP MRI: (T1 weighted contrast images) longitudinal spinal lesions CSF:albumin indicative of blood-CSF barrier dysfunction CSF: ↑monocytes, protein, lactate CT: right axillary lymphadenopathy Infectious/immune panel NSF_2_	*Bh* IgG 1:512 CSF PCR neg Tissue bx finger wound PCR + Recheck *Bh* titers post antibiotics IgG 1:64	Doxycycline × 3 weeks + tapering corticosteroid	Improvement in all clinical signs but residual fatigue, headache, radicular neuropathic pain and mild gait disturbance at 6-month follow-up	[76]
62/F	Cat bite	2 weeks Fever, back pain, inferior limb weakness, hyperalgesia, anuria and fluctuating hypertension	CBC, Chemistry, UA and culture NSF CT: T2 weighted region distal spinal cord CSF: ↑ WBC, protein, ↓ glucose	Not tested	Methylpred, ceftriaxone, vancomycin	No response	[74]
				*Bh* IgG 1:512, IgM 1:160	Doxycycline	Improvement over 4 weeks. Residual pain and weakness in legs	
			MRI: resolution of ATM EMG: ↓motor nerve conduction consistent with GBS	Not repeated	IVIG × 5d	Full recovery by 4th day of therapy	
12/M	Cat bite 5 days prior to fever, back and leg pain	10 days Inability to walk, anuria, fluctuating hypertension	Mildly ↑ platelets, low Hemoglobin, ESR high normal CSF ↓ protein, glucose EMG: evidence of demyelinating polyneuropathy	***Bh*** IgG > 1:320 IgM neg	IVIG	No improvement	[75]
			MRI hyperintense T2 weighted signal, peripheral enhancement and diffusion restriction in spinal cord	Not repeated	Added methypred., rifampin, doxycycline	Near complete resolution after 4d of therapy. Hypoactive deep tendon reflexes otherwise normal at 1-month follow-up	

^1^Urine, blood and CSF culture, CSF latex agglutination, nasal virus culture, plasma regain test, CSF venereal disease panel including enterovirus, HSV and mycoplasma by PCR, EBV, cytomegalovirus, *Mycoplasma pneumoniae*, WNV, *Borrelia burgdorferi*, human T cell lymphotropic virus I/II, CSF ACE, IgG, vitamin B12/folate, ANA, RF, dsDNA antibodies

^2^HSV 1 and 2, varicella zoster virus, cytomegalovirus, EBV, enterovirus, *Borrelia burgdorferi*, *Treponema pallidum*, *Mycoplasma pneumoniae*, tickborne encephalitis virus, *Toxoplasma gondii*, HIV, *Cryptococcus neoformans*, multiplex respiratory viral panel, ANA, ANCA, dsDNA antibodies, antiphospholipid antibodies, onconeural antibody, anti-aquaporin 4 antibodies

*PCR* polymerase chain reaction, *CBC* complete blood count, *MRI* magnetic resonance imaging, *CSF* cerebrospinal fluid, *MBP* myelin basic protein, *NSF* no significant findings, *Bh* *Bartonella henselae*, *CSD* cat scratch disease, *d/c* discontinued, *IVIG* intravenous immunoglobulin, *GBS* Guillain-Barré syndrome, *CRP* C-reactive protein, *CT* computed tomography, *bx* biopsy, *UA* urinalysis, *Methylpred* methylprednisolone, *EMG* electroneuromyogram, *HSV* herpes simplex virus, *EBV* Epstein-Barr virus, *WNV* West Nile virus, *ACE* angiotensin converting enzyme, *ANA* antinuclear antibody, *RF* rheumatoid factor, *HIV* human immunodeficiency virus, *ANCA* anti-neutrophil cytoplasmic antibody

#### Neuroretinitis

Ophthalmological findings in neuroretinitis are characterized by unilateral non-painful vision loss with color discrimination deficits, optic disc edema and a star-shaped pattern of lipid accumulation around the macula, first described as idiopathic stellate maculopathy by Theodor Leber in 1916 [77–79]. Additional ophthalmological findings can include small, white chorioretinal lesions in both the unaffected and affected eye, retinal vasculitis and, occasionally, bilateral vision loss [21, 77]. *Bartonella henselae* is the most common infectious etiological agent associated with neuroretinitis, responsible for about 2/3 of clinical cases [22, 78–89]. Other *Bartonella* species have also been documented, including *B. quintana*, *B. grahamii* and *B. elizabethae*, the latter species first reported in association with Leber’s neuroretinitis, bringing into question an infectious etiology in this condition [86–89] (Table 5). Historically, neuroretinitis associated with *Bartonella* species has fallen under the classification of CSD, in large part due to a predominance of patients reporting interactions with cats. In contrast to typical CSD, which more commonly affects younger children, teenagers or older adults, *Bartonella*-associated neuroretinitis tends to affect individuals in their 30s and 40s and may better be classified as a manifestation of ocular bartonellosis, which includes other presentations such as uveitis, retinal arterial occlusion and Parinaud’s oculoglandular syndrome, characterized by regional lymphadenopathy and ulcerative conjunctival granulomas stemming from trans-conjunctival inoculation of bacteria from infected animal saliva or other sources [90–93]. Other precedent or concurrent clinical signs that may be present in cases of *Bartonella*-associated neuroretinitis include protracted fever, lymphadenopathy, arthralgia, headache and a skin rash. The presenting complaint is most often acutely diminished visual acuity, visual field abnormalities and dyschromatopsia (color blindness). Optic disc edema is a common initial finding, whereas other typical ophthalmological abnormalities, including the development of the macular star, choroidal lesions and vascular occlusion or proliferation, can take several weeks to develop [79–82, 85, 91]. Although pathogenesis of many ocular changes is currently unknown, neovascularization could arise secondary to *Bartonella*’s ability to stimulate vascular proliferation by enhancing vascular endothelial growth factor (VEGF) production upon endothelial cell infection [94–99]. Increased VEGF has been identified for potential to differentiate *Bartonella* infections from other infectious or noninfectious ocular lesions [28]. Ocular inflammation is a common finding in cases of Adamantiades-Behçet’s disease, a systemic condition affecting the microvasculature, and in a publication from 2016, coinfection with *B. henselae* with concurrent elevation in VEGF was documented in a patient suffering from a multitude of wide-spread clinical signs [100, 101].

**Table 5:** Selected cases of *Bartonella*-associated neuroretinitis

CNS neuropathies: neuroretinitis*							
Patient age/sex	Initial symptoms/ clinical findings	Elapsed time to neurological symptoms	Key diagnostic results	*Bartonella* serology/PCR	Treatment/ duration	Outcome	Refs.
13/F	Cat bite Historic postural orthostatic tachycardia	2 months Worsening central vision OD, decreased color vision, pain upon extraocular muscle movement	Slight relative afferent pupillary deficit OD, cecocentral scotoma, visual acuity 20/250 Optic nerve swelling, macular elevation and early star development. Intraretinal edema on OCT Extensive infectious/immune disease NSF_1_	*Bh* and *Bq* IgG and IgM negative (< 1:320) Repeat titers *Bh* positive IgG 1:640	Corticosteroids + doxycycline, rifampin	Return to normal visual acuity and slow recovery of color vision at 3-month follow-up	[85]
60/F	Flu-like illness: headache, chills, fatigue	4–6 weeks Non-painful vision loss OS that was improving by the time of ophthalmological exam	OS sectoral edema inferior temporal region and partial stellate exudative pattern macular region, afferent pupillary defect, visual acuity 20/40. Intraretinal edema on OCT Infectious and immune disease testing NSF_2_	*Bh* neg *Bq* IgG > 1:1024 IgM neg	No therapeutics	Clinical resolution after 4 months	[86]
55/F	Acute onset headache, irritability and anxiety	1 year Progressive decrease in visual acuity leading up to evaluation	Visual acuity OD 20/125, OS 20/50, OU macular edema, retinal vasculitis, vitreitis, papillitis and posterior synechiae Elevated ANA, ESR, CSF pleiocytosis_3_	No initial testing	Corticosteroids, acetazolamide	Improved visual acuity and mental condition, no change to ocular inflammatory parameters	[87]
			Macular edema, sporadic atrophic scars in peripheral retinas, fluorescein angiogram consistent with neuroretinitis Repeat infectious/immune disease testing_4_	EIA *Bh* IgG 1:1000 *Bh* IgG 1:64 IgM neg PCR + *Bg* ocular fluid	Rifampin + doxycycline × 4 weeks	Resolution of inflammatory disease	
				Repeat *Bh* titer post antibiotic 1:32. Repeat PCR *Bg* neg		Cataract development surgically corrected	
31/M	Racoon bite	4–6 weeks Acute decrease in visual acuity	Optic nerve swelling OD. Brain MRI NSF. ESR, ANA, RPR NSF. CBC ↑WBC (neutrophilia)	No initial testing	Methylpred. IV × 3 days	Worsening of visual acuity	[88]
					IVIG, Rabies vaccine	Macular star development	
			Optic disc swelling, focal white infiltrates sub- and intra-retinal and proximal to optic nerve. Grade 1 afferent pupillary defect Resolving macular star Acuity counting fingers Infectious disease testing_5_	*Bh* and *Bq* serology neg *Be* serology + IgG 1:64	Erythromycin × 4 weeks	Repeat *Bartonella* serology: *Bh*, *Bq* neg, *Be* ↑ 1:128	
					Extended course to 6 weeks	*Be* titer neg. 1:32 6-month follow-up: visual acuity 20/200, grade 2–3 afferent pupillary defect, marked optic disc pallor, resolving retinal exudates	

^*^Selection of case reports chosen to represent clinical spectrum

^1^Meningitis PCR testing of CSF (excluded *Bartonella*) negative, CSF culture negative, no specific oligoclonal bands. Toxoplasma and Lyme PCR negative. HIV, *Treponema pallidum* serology negative. ACE, anticardiolipin, ANA, ANCA all NSF

^2^Lyme (*Borrelia burgdorferi*) titers, ACE, RPR, FTA-ABS, ANA all NSF

^3^CBC, blood chemistry, HLA-B27 normal. HSV, VSV, *Borrelia burgdorferi* NSF. CSF testing for HSV, VZV, *B. burgdorferi* and enteroviruses neg

^4^Repeat ESR, CBC NSF. ACE, *Treponema pallidum*, *Borrelia burgdorferi*, HIV neg. Thoracic radiograph NSF

^5^*Toxoplasma gondii* IgG + , neg. IgM. Toxocara + 1:16. Rabies titer neg. Tuberculin skin test neg

*OD* right eye, *MRI* magnetic resonance imaging, *CSF* cerebrospinal fluid, *OS* left eye, *OCT* optical coherence tomography, *NSF* no significant findings, *Bh* *Bartonella henselae,* *Bq* *Bartonella Quintana,* *ACE* angiotensin converting enzyme, *RPR* rapid plasma regain test for syphilis, *FTA-ABS* fluorescent treponemal antibody absorption, *ANA* anti-nuclear antibody, *OU* both eyes, *ESR* erythrocyte sedimentation rate, *EIA* enzyme-linked immunoassay, *PCR* polymerase chain reaction, *Bg* *Bartonella grahamii*, *CBC* complete blood count, *HSV* herpes simplex virus, *VSV* varicella zoster virus, *ACE* angiotensin-converting enzyme, *HIV* human immunodeficiency virus, *WBC* white blood cells, *Methylpred* methylprednisolone, *IVIG* intravenous immunoglobulin, *Be* *Bartonella elizabethae*

Clinically, most cases resolve without intervention; however, antimicrobial drugs, corticosteroids and intravenous immunoglobulin have been utilized, especially when the patient has other systemic signs of bartonellosis. In a multicenter retrospective review by Chi et al. (2012), it was noted that good visual acuity at presentation and the absence of systemic symptoms were the only factors associated with good visual prognosis; use of systemic antibiotics or corticosteroids had no association [83]. Similarly, in a 2018 review by Abdelhakim and Rasool, the authors agree that in cases of *Bartonella*-associated neuroretinitis, visual recovery is favorable regardless of medication administration [21]. Early antimicrobial intervention has been suggested, however, to hasten ocular recovery, eliminate the inciting pathogen and reduce potential sequelae of chronic infection [81]. Of note, there is a solitary case report by Rodriguez et al. in which a 28-year-old woman with a history of systemic lupus erythematosus treated with immunosuppressive drugs developed neuroretinitis in combination with multiple brain abscesses, with *B. henselae* detected via PCR from a brain biopsy [102]. It was hypothesized that hematogeneous spread of the organism resulted in this pathology [102].

Although most cases of neuroretinitis appear to occur following contact with a cat, there are several published cases in which either cat interaction did not occur or there was association with a different animal species [103]. In Table 5, a patient suffering from a racoon bite was diagnosed with neuroretinitis caused by *B. elizabethae*. This species was first reported in a patient with endocarditis in 1993, later found in association with IV drug use, and was recently detected in an immunocompromised patient with bacillary angiomatosis [104, 105]. Rats are the suspected mammalian reservoir for *B. elizabethae*, although it has also been found in association with the murine genus *Mastomys* [106]. A patient with *B. grahamii* DNA isolated from her ocular fluid had suffered not only from neuroretinitis, but also acute onset headache and behavioral changes, including anxiety and irritability, signs that are more commonly seen with *Bartonella*-associated encephalitis/encephalopathy [87]. *Bartonella*-associated neuroretinitis has also been reported in association with bull ant stings [107], and bites or scratches from a pet dog, ferret and guinea pig [103, 104, 108], as well as multiple cases in which cat or animal contact did not occur, highlighting the need for clinical suspicion even in the absence of animal contact [103, 109, 110].

#### Encephalitis and encephalopathy

Encephalitis denotes inflammation within the brain whereas encephalopathy encompasses a range of symptoms involving altered mental status, consciousness or personality that can occur in the absence of inflammation. These topics will be covered jointly in this section because of the interchangeable use of the terminology in the reported literature, reports of *Bartonella*-associated encephalitis progressing rapidly to encephalopathy and reports in which encephalopathy was not associated with inflammation [111].

Classically, infectious encephalitis can be caused by numerous pathogens, including viruses, bacteria, fungi and others, although etiology remains unidentified in up to 63% of cases [24, 111–121]. Due to the potential for high mortality, it is imperative that potentially treatable infectious causes be rapidly addressed, both diagnostically and therapeutically [24, 112, 113]. Herpes simplex virus (HSV) is cited as the most common infectious cause of encephalitis in Western countries, but worldwide, Japanese encephalitis virus is the most prevalent cause [113]. In meningoencephalitis, inflammation involves the brain parenchyma as well as the surrounding meningeal membranes [113]. In terms of *Bartonella*-associated disease, encephalitis occurs in 1–7% of cases of classical cat scratch disease, with children most commonly impacted [114–118]. Symptoms generally include headache and acute onset of seizures, often classified as status epilepticus refractory to treatment [115, 119]. Additional encephalopathic signs include lethargy, confusion, disorientation, sleep disorders, expressive aphasia and word-substitution errors. Personality changes such as agitation and combative or aggressive behavior are also fairly common (40% of cases) [116]. Other reported comorbid symptoms include gait abnormalities, dystonia, weakness, myelitis and hemiplegia, indicating that a meningeal component may be more commonly encountered [116, 117, 119, 120]. Concurrent signs of gastrointestinal pain are not uncommon, and in most reported cases, lymphadenopathy and fever are also present, accentuating the systemic nature of infection [119, 120]. Similar to other sources of encephalitis, brain imaging is generally normal despite neurological deficits, although subtle changes may occur [121, 122].

*Bartonella henselae* is the most commonly reported species identified in cases of *Bartonella*-associated encephalitis/encephalopathy, although case reports exist where other species or co-infections have been reported. Regarding *B. henselae* infections, most cases are in children aged 7 through 12 [123], and hospitalization is more common in males [123]. Prior interactions with cats are common, and most patients have preexisting fever 1–2 weeks prior to the onset of neurological signs [123]. The most common presenting abnormality is seizures (status epilepticus), while electroencephalograms (EEG) are consistent with encephalopathy (slow delta waves and lack of epileptiform discharges) [113]. Table 6 contains select cases in children to demonstrate the clinical spectrum of *B. henselae*-associated encephalitis [120, 124–126].

**Table 6:** Selected cases of *Bartonella*-associated encephalitis and encephalopathy

CNS neuropathies: encephalopathy and encephalitis due to *Bartonella henselae* infection*							
Patient age/sex	Pertinent medical history	Neurological and related symptoms	Key diagnostic results	Bartonella serology/PCR	Treatment/ duration	Outcome	Ref.
11/F	Oppositional defiant disorder, sensory integration disorder	Seizure following head trauma Continued post-ictal confusion, agitation, tachycardia	Baseline diagnostics all NSF_1_ aside from mild ↑ glucose EKG: sinus tachycardia EEG: slow/moderate amplitude delta waves bilateral, more prominent anteriorly. No interictal epileptiform discharges or sharp wave complexes	Not initially evaluated	Single dose benzodiazepine	Return to baseline status over a few days, mild gait instability	[124]
		Recurrent seizures, new fever and hypersomnolence developed 1 day post hospital discharge	Hypoxia during seizure episode. No change to EEG	Not initially evaluated	Levetiracetam	agitation, nausea/vomiting, headaches, truncal instability, poor balance	
			Repeat MRI NSF Additional diagnostic testing NSF_2_ aside from eosinophilia	*Bh* IgG > 1:1024, IgM < 1:16	Doxycycline + rifampin × 14 days	Return to baseline function by 3-month follow-up, despite d/c doxycycline due to vomiting	
7/M	Fever 1 week prior to neurological event	Acute onset seizure, loss of consciousness, hemiparesis and difficulty breathing	CSF, cerebral CT NSF. EEG basal activity slow Extensive infectious/immune panels_3_	Not initially evaluated	Not described	Confusion and visual hallucinations	[120]
		Recurrent fever	Involuntary orolingual movements. Persistent hallucinations	Pending	Ceftriaxone + clindamycin × 5 days	Overall improvement	
					Empiric azithromycin	Discharged	
		Recurrent seizures	Further diagnostics_4_ Echocardiogram: mild TI EEG: quick diffuse activity Ophthalmology exam: blurring of discs OU, normal acuity, progresses to effacement of disc OU. Anisocoria with slight prominence of OS. CSF ↓ glucose AUS: mild hepatosplenomegaly	IFI + *Bh*	Ciprofloxacin, cotrimoxazole + rifampicin	Diplopia and persistent headache	
				*Bh* IgG 1:256 IgM > 1:20 Blood and CSF PCR +	Azithromycin + rifampin × 4 weeks	Remission of neurological and fundic abnormalities Resolution by 6-month follow-up. Repeat titers neg	
11/F	Malaise, right hip pain, right lower quadrant pain, intermittent fever 2 weeks prior to neurological event	Acute onset seizure	Mild ↑WBC and ↑ESR prior to seizure episode Recheck CBC: ↑WBC, platelets, ESR, CRP. CT of head NSF. CSF NSF Infectious disease screening_5_ Focal ultrasound right lower quadrant- lymphadenopathy	Not initially evaluated	Empiric vancomycin, ceftriaxone + acyclovir Fosphenytoin	Occasional slurred speech, word-finding difficulty and short- term memory loss	[125]
				*Bh* IgG 1:1024	Azithromycin + doxycycline × 7 days	Rapid improvement to baseline neurological status	
12/F	Fever	1 day later acute onset altered mental status, confused speech, pain in extremities	Mild ↑WBC with bandemia. Strep A pos._6_ CT NSF	Pending	Vancomycin + ceftriaxone + acyclovir Levetiracetam	Next day flaccid paralysis left arm, urinary incontinence	[126]
			MRI NSF aside from paranasal sinusitis EEG: slowing in right hemisphere Blood, urine and CSF cultures NSF Infectious/immune diagnostics_7_	*Bh* + 1:2560	Doxycycline × 14 days Tapered off Levetiracetam	Complete recovery by 1 week follow-up	

^*^Selection of case reports chosen to represent clinical spectrum

^1^CBC, metabolic panel, magnesium, urinalysis and urine toxicology screen, head/neck CT, brain MRI, CSF encephalitis/meningitis panel, blood and urine cultures all NSF

^2^Extensive infectious disease testing, liver function, blood and CSF cultures NSF; cultures negative for Bh. No evidence of optic neuritis

^3^HSV, VSV, HIV, Enterovirus neg. Toxicology neg. Thyroid normal. Cerebral MRI and thoracic radiograph NSF. CD4:CD8 lymphocytes normal. IgM, IgG, IgA, C3, C4 normal

^4^Anti-receptor antibody for N-methyl-D-aspartate. Repeat brain MRI NSF

^5^EBV, HSV, Enterovirus PCR from CSF. Urine toxicology neg. Nonspecific lymphadenopathy lower right quadrant, no joint effusion R hip. VSV, WNV, arborvirus panel, *Ehrlichia* neg

^6^Rapid strep test pos. with no tonsillitis or throat erythema, consistent with carrier status. Metabolic panel, UA and urine drug screen, CSF NSF

^7^HSV, VZV neg. (CSF). WNV, EBV, Arborvirus neg. *Mycoplasma pneumoniae* elevated IgM (1:1861) with neg. PCR. Anti-streptolysin and anti-DNAseB antibodies elevated

*NSF* no significant findings, *EKG* electrocardiogram, *EEG* electroencephalogram, *MRI* magnetic resonance imaging, *Bh* *Bartonella henselae,* *d/c* discontinue, *CSF* cerebrospinal fluid, *CT* computed tomography, *TI* tricuspid insufficiency, *OU* both eyes, *OS* left eye, *AUS* abdominal ultrasound, *IFI* indirect immunofluorescence, *PCR* polymerase chain reaction, *WBC* white blood cells, *ESR* erythrocyte sedimentation rate, *CBC* complete blood count, *CRP* C-reactive protein

A thorough patient history can provide critical clues in forming differential diagnoses and help to avoid overlooking treatable conditions. Özer (2021) underscores the importance of obtaining a detailed history in a case report involving a 3-year-old girl referred for a suspected neurodegenerative metabolic condition [127]. The child was suffering from an impaired ability to walk, hand tremors and irritability, and had previously been treated medically for a cat scratch and subsequent fever, lymphadenopathy, otitis and mastoiditis. The neurological signs occurred about 6 weeks after a 1-week course of amoxicillin-clavulanate prescribed for the febrile illness. Treatment for neurobartonellosis was delayed because CSD, the preliminary diagnosis, was considered self-limiting by the infectious disease department of the admitting hospital. Extensive metabolic, infectious and immune diagnostics, CSF testing and imaging were performed both before and after titers for *B. henselae* were obtained and treatment initiated with rifampin. The author emphasizes that CSD is not considered a differential diagnosis in cases of serious neurological illness and is misclassified as “self-limiting and benign,” a conclusion that given the broad spectrum of *Bartonella*-associated illnesses bears reconsidering [127].

Encephalopathic symptoms, such as behavioral changes or psychiatric symptoms, may be the predominant clinical feature in some patients. In a case report by Bejarano et al. (2020), a 2-year-old boy presented with seizures and impaired consciousness following a traumatic head injury [119]. Rapid deterioration was noted, and the patient was placed on broad-spectrum antimicrobials and acyclovir along with anticonvulsants for refractory generalized and focal seizures. The patient also experienced hypotonia, incoordination, disorientation and hypersomnia. As there was no history of cat contact, *Bartonella* titers were not obtained until 14 days after admission. In the interim, the boy was treated with multiple anti-epileptic drugs with medication changes due a lack of efficacy or suspected potential adverse drug reactions. Psychiatric symptoms including hallucinations and unprovoked laughter were noted. Several behavioral symptoms, including aggression, impulsivity and restlessness, persisted despite successful control of the seizure activity once rifampin and azithromycin were instituted for neurobartonellosis. Additionally, this patient experienced recurrent neurological symptoms, including ataxia, hypersomnia and incomprehensible language that occurred 2 days after the cessation of his antibiotic course. An EEG displayed diffuse brain involvement, whereas MRI documented only mildly restricted contrast diffusion to the left cerebral cortex, consistent with a history of recent seizures. Rapid recovery accompanied reintroduction of antimicrobial therapy. At the patient’s 9-month follow-up appointment, he had experienced no further seizure activity while continuing anti-convulsant medications, but impulsivity, aggression and agitation persisted, along with the development of significant hyperactivity [119]. Although many cases of *Bartonella*-associated encephalitis/encephalopathy report some degree of neuropsychiatric symptomology, this case is especially interesting because of the youthful age of the patient and the continued symptoms following antibiotic treatment [119]. Although immune-mediated encephalitis secondary to the initial infection remains an etiological consideration, persistent, incompletely treated infections are also possibilities. Cases with prominent neuropsychiatric signs will be discussed in a following section.

Species other than *B. henselae*, including *B. quintana* [128, 129], *B. washoensis* [130] and *Bartonella* coinfections have been noted in cases of encephalitis/encephalopathy [131] (Table 7). The agent responsible for trench fever, *B. quintana*, is transmitted by the human body louse (*Pediculus humanus humanus*), and humans are its natural reservoir [132, 133]. In addition to the louse, *B. quintana* DNA has been isolated from dental pulp from cats, cat and monkey fleas, and a woman following a cat bite [133–136]. Endocarditis is the most common presentation, and *B. quintana* is responsible for 75% of *Bartonella*- associated endocarditis cases [136]. Neurological manifestations have been less commonly reported. In a case report from France, a previously healthy 20-year-old woman presented with a 5-day history of fever and headache [128]. Viral encephalitis was initially diagnosed based upon elevated CSF lymphocytes and an elevated blood CRP level. She was not seroreactive to *B. henselae* but was *B. quintana* seroreactive at the lowest positive dilution (IgG 1:50). Continued headache compounded by agitation and confusion developed over 3 days, at which point reevaluation of her *Bartonella* titers documented a rising titer to *B. quintana* (Table 7). She was treated with IV ofloxacin and was normal at a 1-year follow-up appointment [128]. *Bartonella quintana* has been implicated in two additional cases of encephalitis in children, in which *Bartonella* coinfections were present [129]. The first case was more typical in presentation: an 8-year-old boy with a history of kitten interaction presented with lymphadenopathy and fever. Seizures developed a few weeks after he had been treated with an antibiotic for typical CSD, at which point *B. henselae* titers were determined to be seroreactive (Table 7). Phenytoin was prescribed for seizure activity, and no additional antibiotics were given. The boy developed cognitive decline, ataxia, tremor and agitation 2 weeks later, at which point rising titers to *B. henselae* and amplification of *B. quintana* DNA from the patient’s CSF were indicative of *Bartonella*-associated encephalopathy. The anti-seizure medication was switched to carbamazepine, and the patient improved over a 2-week period (Table 7). In a second case, a young girl had extensive, chronic neuro-behavioral and neurological conditions diagnosed over a period of several years (Table 7) [131]. Historically, she had evidence of normal to low white blood cells in the face of relapsing fevers. Extensive prior testing for infectious and immune-mediated conditions were negative. As part of a study evaluating healthy and ill people for evidence of *Bartonella*, *B. quintana* exposure was first diagnosed by our laboratory via serology [131]. Despite treatment with a combination of antibiotics for a 2-month period, there was minimal symptomatic improvement. Repeat serology was seronegative to several species of *Bartonella*, but PCR detected DNA from *B. quintana* (blood) and *B. vinsonii* subsp. *vinsonii* (enrichment blood culture). Typically associated with rodents, *B*. *vinsonii* subsp. *vinsonii* was first reported in association with a febrile illness in Thailand [137]. The chronicity of clinical symptoms in this case may be indicative of a protracted infection. Whether infection persistence or coinfection with a second *Bartonella* species impacted the lack of clinical response remains unknown. In a third case, a 47-year-old woman with extensive animal exposure developed symptoms concerning for meningitis or early sepsis but failed to fully respond to several antimicrobial agents [130]. *Bartonella washoensis* was documented in her blood cultures using multiple *Bartonella* spp. gene targets. *Bartonella washoensis* has been isolated from ground squirrels and their fleas. Although specific animal scratch or bite wounds were denied, the patient had a history of handling a squirrel carcass, and this combined with the presence of squirrel burrows on her property potentially impacted her *Bartonella* exposure [130]. The only other case of *B. washoensis* infection involved a 70-year-old man with fever and myocarditis [138]. There is abundant literature reporting encephalitis in patients with coinfections with *Bartonella* and other pathogens, particularly *B*. *burgdorferi* and *Toxoplasma gondii* [43, 139, 140]. Coinfections are beyond the scope of this review.

**Table 7:** Encephalitis and encephalopathy cases secondary to alternate *Bartonella* species or coinfections

CNS neuropathies: encephalopathy and encephalitis due to diverse *Bartonella* species and coinfections							
Patient age/sex	Pertinent medical history	Neurological and related symptoms	Key diagnostic results	*Bartonella* serology/PCR	Treatment/ duration	Outcome	Ref.
20/F	No prior history of illness	Fever, headache, stiff neck × 5 days	CSF ↑WBC, 78% Lymphocytes, ↑albumin ↓glucose ↑ CRP Meningococcus neg	*Bh* neg *Bq* + IgG 1:50	Supportive care for suspected viral encephalitis	Initial improvement to hospital discharge. 3d later presented with recurrent fever, headache, confusion and agitation	[128]
			CSF WBC higher than previous with 90% lymphocytes, ↑albumin, normal glucose Infectious disease screening NSF_1_	*Bh* neg *Bq* + IgG 1:400	IV Ofloxacin × 7 days	Resolution of fever within 48 h, with complete remission at 1-year follow-up	
8/M	Right axillary lymphadenitis, fever History of kitten scratches	None	None	Suspected CSD	Cefadroxil × 10d	Resolution of symptoms	[129]
		cluster seizures with prolonged post-ictal period 3 weeks post antibiotics	EEG: generalized slowing with no epileptiform activity, CT normal, CSF 1 RBC/mm3, slightly ↑glucose	*Bh* + EIA IgG 46, IgM 18	Phenytoin	Readmitted at 2 weeks post-discharge for worsening signs	
		2 weeks post seizures, developed agitation, cognitive and memory decline, ataxic gait, intention tremor	EEG: mild slowing of posterior dominant rhythm with no epileptiform activity MRI NSF. CSF: ↑protein, negative gram stain and culture	*Bh* + IgG 63, IgM neg *Bq* PCR + (CSF)_2_	Phenytoin discontinued; carbamazepine instituted	Improvement with normal mental status by 2-week follow-up, *Bh* serology dropped to IgG 15 (slight positive) and IgM < 12 (neg), *Bq* serology not obtained	
47/F	1 day history of fever, chills, headache, nausea, vomiting, abdominal pain	Progression over a few hours to severe headache, photophobia, bilateral joint pain upper and lower limbs	Cranial and abdominal CT, thoracic radiographs NSF Low normal HCT Normal WBC count but high % neutrophils, low % lymphocytes CSF normal except 1 RBC and 6 WBC/mm^3^_3_	Blood cultures pending	Empirical vancomycin + chloramphenicol, switched for aztreonam	Clinical improvement with low normal HCT, slightly low WBC and platelets Discharged after 3d on moxifloxacin	[130]
				Aerobic bacterial growth, suspected *Capnocytophaga*	Levofloxacin × 10 days	6-week follow-up leucuria, antibiotic switched because of presumptive UTI	
				*16 s rRNA*, *groEL* and *gltA* PCR 99.6–100% identity to *Bw*._4_	No further treatment mentioned	8-week follow-up persistent muscle and bone pain	
14/F	Extensive illness over 5-year period_5_	Neurological diagnoses included ADHD, migraines, auditory processing disorder, learning disability and myalgic encephalomyelitis	Historic ↓WBC in face of fever, normal chemistry and UA Extensive infectious/immune testing_6_	*Bq* + 1:64	Doxycycline, rifampin, clarithromycin × 2 months	Minimal improvement	[131]
				Serology neg. to *Bq*, *Bh*, *Bvb* I-III, *Bk* PCR + *Bq*, *Bvv*_7_	No further treatment mentioned	Lost to follow-up	

^1^EBV, CMV, WNV, HIV, *Rickettsia conorii*, *Rickettsia typhi*, *Chlamydia pneumonia*, *Brucella* Neg. Normal echocardiogram

^2^*Bartonella* *quintana* sequencing > 99% homology to a segment of the citrate synthetase gene

^3^CSF culture and *Enterovirus* PCR neg. Blood chemistry, coagulation, liver function NSF

^4^*Bartonella* genes screened based on bacterial properties of peptidase activity and gas chromatography of fatty acid esters. Genetic sequence identity confirmed through NCBI Blast tool. *gltA* sequence 100% identity to a sequence obtained from a California ground squirrel. Bootstrap analysis of *gltA* and *groEL* clustered with other *B. washoensis* strains. Fleas obtained from ground squirrel and burrow sites on patient’s property were *gltA* PCR and sequence positive for two *Bartonella* strains, *B. washoensis* strain NVH1 (obtained from a patient with fever and myocarditis) and *B.* species strain Sb1659nv from a California ground squirrel. Final diagnosis is *Bw*-like Bartonella infection

^5^Intermittent fever, ocular pain, blurred vision, balance issues, headaches, irritability, confusion, disorientation, hallucinations, memory loss, anxiety, panic attacks, tremors. Fatigue, multiple joint pain, muscle weakness and myalgia, tachycardia, diarrhea. Frequency and number of symptoms progressively increased

^6^Further medical testing revealed dairy, gluten and oak allergy, *Clostridium difficile* colitis. IgA, IgM, IgE, IgG normal. Extensive viral, protozoal, bacterial, including *Borrelia burgdorferi*, all neg. or inconclusive. *Mycoplasma pneumoniae* IgG and Coxsackie A/B antibody titers intermittently increased

^7^ITS PCR and sequencing for *B. quintana* (from blood) and *B. vinsonii* subsp. *vinsonii* (from culture-enriched blood). *Bvv* shared 100% identity with the Baker isolate strain from Quebec, Canada, but not with multiple rodent isolates

*CSF* cerebrospinal fluid, *WBC* white blood cells, *CRP* C-reactive protein, *Bh* *Bartonella henselae*, *Bq* *Bartonella quintana*, *NSF* no significant findings, *IV* intravenous, *EEG* electroencephalogram, *CT* computerized tomography, *RBC* red blood cell, *EIA* enzyme immunoassay, *MR* magnetic resonance imaging, *PCR* polymerase chain reaction, *HCT* hematocrit, *PMN* polymorphonuclear cell/neutrophil, *UTI* urinary tract infection, *16 s* *rRNA* 16 s subunit of ribosomal RNA, *groEL* heat shock protein gene, *gltA*- citrate synthase gene, *Bw* *Bartonella washoensis*, *ADHD* attention deficit hyperactivity disorder, Bvb I-III- *Bartonella vinsonii* subsp, *berkhoffii* types I, II, III, *Bk* *Bartonella koehlerae*, *Bvv* *Bartonella vinsonii* subsp. *vinsonii*, *EBV* Epstein-Barr virus, *CMV* cytomegalovirus, *WNV* West Nile virus, *HIV* human immunodeficiency virus, *NCBI* National Center for Biotechnology Information, *ITS* 16 s-23 s rRNA intergenic transcribed spacer sequence

#### Cerebral vasculitis and aneurysm

Similar to peripheral vasculitis, inflammation in brain vasculature results in blood vessel damage. This can be primary, limited to the brain, meninges and spinal cord, or secondary to systemic vasculitis [141]. Headache is the most common clinical finding, often with sub-acute onset, followed by a variety of neurological/neuropsychiatric features including behavioral or personality changes, cognitive dysfunction and dementia. Transient ischemic attacks (TIA) occur in up to 50% of people with CNS vasculitis, and less common symptoms can include seizures, cranial neuropathies, ataxia and coma [141]. Concurrent signs of illness, such as fever, may be present when central vasculitis is secondary to a systemic condition. In these cases, central vasculitis tends to occur later in the disease process, which may explain delayed onset or progressive symptoms [141]. Importantly, the effects of various pathogens can “mimic” vasculitis and may impact any size of blood vessel, compounding the difficulty of etiological determination in these cases [142, 143]. *Bartonella* has rarely been diagnosed in cases of cerebral vasculitis (Table 8). Notably, the patients displayed vastly different clinical presentations, with one patient displaying acute onset headaches and other neurological signs, while the other suffered a protracted condition spanning several years [143, 144].

**Table 8:** Cases of cerebral vasculitis secondary to *Bartonella* infection

Cerebral vasculitis associated with *Bartonella* infection								
Diagnosis	Age/ sex	Medical history	Neurological symptoms	Pertinent diagnostics	Bartonella serology/ PCR	Treatment/ duration	Outcome	Ref.
Cerebral vasculitis	60/F	3 weeks thunderclap headaches, photo and phonophobia, nausea and vomiting	Brief episode of slurred speech, expressive aphasia, right facial droop and right hemi-paresis	Initial brain CT/MRI NSF. DSA: intracranial med. + lg. vessel narrowing + fusiform dilatations. IVWI: multifocal concentric vessel wall thickening + enhancement_1_	Pending	Empiric high dose IV steroids	Resolution of all symptoms	[143]
					*Bartonella* IgM 1:256 IgG neg	Doxycycline + rifampin + oral steroids	4-week follow-up MRI improvement. Decreasing titers (IgM 1:80)	
Cerebral vasculitis and infarction	11/F	Flu-like illness night sweats, abdominal pain, bloating and constipation	Sudden onset headaches, difficulty walking, ataxia and left-sided paraparesis	MRI: large, focal demyelinating mass right parietal lobe. Biopsy: vasculitis, cerebral infarction_2_	Not initially tested	High dose IV steroids for presumptive autoimmune disease	Multiple ensuing diagnoses; idiopathic vasculitis, GBS, MS, ADEM over 3 years	[144]
		Non-febrile respiratory illness, chest pain	3 weeks later headaches anxiety, ocular floaters, depression, fatigue, visual and auditory hallucinations	No additional diagnostics noted		No additional treatment noted	1-year period of time elapsed to neurocognitive signs	
		No other episodic fever or other signs noted	Neurocognitive dysfunction, left-sided paralysis, hemianopia, seizures, dysphagia, laryngitis and severe confusion	No additional diagnostics noted		IVIG	Recovery over several months; new allergic reactions to pork, lactose, gluten and corn	
		No other inciting episodes noted	Recurrent progressive paresis 5 years after last episode	IgA, IgM, IgG low		IVIG, fluconazole	Status epilepticus requiring medically induced coma	
			Tetraplegia, dysphasia, severe facial palsy upon coma recovery	Equivocal Lyme on Western blot		Penicillin added to treatment plan above	Rapid improvement in all neurological signs	
			Overall deterioration requiring physical support and full-time care_3_	*Bh* visualized in FFPE brain tissue	IFA neg *Bh* + PCR/ sequence_4_ Repeat *Bh* testing neg_5_	Atovaquone, azithromycin + ceftriaxone	Dramatic improvement in all clinical signs	
						Ceftriaxone, metronidazole, azithromycin × 9 weeks	Recurrent seizure and cognitive decline then slow improvement over 7 months	

^1^CSF and transcranial Doppler study NSF. Infectious and inflammatory screening neg

^2^Surrounding perivascular lymphoplasmacytic vascular infiltration + sparse hemosiderin in small arteries and venules. Perivascular lymphocytes, primarily T cells, scattered B cells. MIB1 minimal, no EBV immunoreactivity

^3^Neurobartonellosis, babesiosis and Lyme suspected based on clinical presentation

^4^IFA for Bh strains San Antonio 2 and Houston 1, *B. vinsonii* subsp. Berkhoffii genotypes I–III, *B. koehlerae* all negative. Positive PCR and genetic sequencing for Bh (99% identity NCBI) from blood. PCR + from FFPE brain tissue

^5^Negative IFA and PCR as above, from blood, serum and post-BAPGM enrichment blood culture

*CT* computerized tomography, *MRI* magnetic resonance imaging, *NSF* no significant findings, *DSA* digital subtraction angiography, *IVWI* intracranial vessel wall imaging, *IV* intravenous, *MIB1* Mindbomb E3 ubiquitin protein ligase 1 minimal staining, Epstein-Barr virus negative, *GBS* Guillain-Barré syndrome, *MS* multiple sclerosis, *ADEM* acute disseminated encephalomyelitis, *IVIG* intravenous immunoglobulin, *Ig* immunoglobulin, *Bh* *Bartonella henselae,* *FFPE* formalin-fixed paraffin embedded tissue, *Bh* *Bartonella henselae*, *IFA* immunofluorescent antibody, *PCR* polymerase chain reaction

Another vascular condition, intracranial infectious aneurysm, is considered a rare cause of cerebral aneurysm development [145]. Pathogenically, these occur secondary to infection where neutrophilic vasculitis leads to destruction of the vascular elastic lamina, subsequently causing vascular weakening and ballooning [145]. Bacterial pathogens are the most prevalent cause of mycotic aneurysms, and *Bartonella* has been associated with cerebral aneurysm and embolism as a sequela to culture-negative endocarditis [145–149]. *Bartonella* is one of the most common causes of culture-negative endocarditis, and several species have been implicated, most commonly *B. henselae* and *B. quintana* [150]. Interestingly, approximately 30% of culture-negative endocarditis cases demonstrate neurological symptoms as the initial clinical presentation [147]. *Bartonella* endocarditis has a higher prevalence in middle-aged men and a predilection for the aortic valve [147]; however, a diversity in valvular locations and symptoms can occur. Table 9 summarizes cases in which neurological symptoms secondary to aneurysm preceded the diagnosis of *Bartonella*-associated endocarditis [146–149].

**Table 9:** Select cases of cerebral aneurysms secondary to occult *Bartonella* infections with neurological symptoms as primary presentation

Cerebral aneurysm related to *Bartonella*-associated endocarditis presenting with primary neurological signs							
Age/ sex	Clinical history	Neurological signs	Pertinent diagnostics	*Bartonella* serology/PCR	Treatment/duration	Outcome	Ref.
48/M	Syncope	Altered sensorium	CT: large intracranial hemorrhage. CCA: fusiform aneurysm TTE: mod MR with thickened MV, no vegetative growth. Blood cultures neg	Not initially indicated	Craniotomy + endovascular embolization	Headaches developed after 2 years	[146]
		New onset headaches	CT: Intraventricular hemorrhage. CCA: new left distal P3 aneurysm. TTE: myxomatous change to mitral valve. Immune-mediated necrotizing GN Blood cultures and infectious disease screening neg_1_	*Bh* IgG 1:2560	Repeat endovascular embolization Doxycycline + rifampin	Readmission 2 months after discharge for CHF	
		No new signs	CCA: new right middle cerebral aneurysm	*Bh* + PCR_2_	+ gentamicin, aneurysm surgery, valve replacement	Improvement on 3 months of doxycycline	
39/M	↑BP Intermittent fever	Tonic-clonic seizure	CT: left parietal intraparenchymal hemorrhage, subdural hematoma, transtentorial + uncal herniation suspected. CTA: hyperattenuated focus consistent with aneurysm, also secondary unruptured aneurysm. Clot culture neg	Not initially indicated	Hemicraniotomy and clot evacuation Vancomycin + meropenem	Developed fever with negative blood and urine cultures, responded to cefazolin	[147]
			ESR and CRP ↑ TTE: 2 small mobile MV vegetations		Vancomycin, cefepime + gentamycin	Intermittent fevers developed post-op	
			Blood culture neg Immune panel neg *Coxiella burnettii* neg	*Bh* 1:1024	Gentamycin × 2 weeks doxycycline × 6 weeks	Aneurysm stabilization Non-verbal, limited movement, feeding tube dependent at 6 months	
42/M	↑BP, lipid DM	Acute onset aphasia, weakness in extremities	MRI: non-hemorrhagic infarct left middle cerebral artery. MRA confirmed absence of blood flow. TTE: suspected vegetations aortic valve Blood culture neg._2_	Submitted	Empiric vancomycin + ceftriaxone	Continued doxycycline × 12 months Improvement and stabilization lead to outpatient rehabilitation	[148]
				*Bh* IgG > 1:2560	Gentamicin + doxycycline × 2 weeks		
60/M	Flu-like illness 1 months prior	Acute headache, right upper extremity tingling and numbness	CT: left basilar subarachnoid hemorrhage, CTA left middle cerebral artery aneurysm TTE: MR, valvular vegetations Blood cultures neg_3_	*Bh* IgG > 1:1024	Endovascular embolization Vancomycin + ampicillin/sulbactam	Discharged on IV gentamicin + oral doxycycline × 2 weeks, repeat CTA in 2 weeks	[149]
			Repeat CTA: new left middle cranial artery aneurysm Repeat TTE: MR worse, MVP with increased size vegetations	*Bh* PCR + (valves)	Endovascular embolization Mitral valve replacement	Repeat 2 weeks gentamicin + 6 weeks doxycycline	

^1^*Brucella, Coxiella* and *Bartonella quintana* neg

^2^PCR positive and genetic sequence obtained from aneurysm: PCR positive from valve

^3^Slightly low hemoglobin, HCT, MCV, MCH. Mild ↑ immature granulocytes. ↑ d-dimer. Mild ↓ sodium. Mild ↑ creatinine. Mild ↓ TP and albumin. ↓ LDL and HDL. ↑ B12. *Bq*, *Coxiella*, *Treponema pallidum* and SARS-CoV2 neg

*CT* computed tomography, *CCA* coronary angiogram, *TTE* transthoracic echocardiogram, *MR* mitral regurgitation, *MV* mitral valve, *GN* glomerulonephritis, *Bh* *Bartonella henselae,* *CHF* congestive heart failure, *PCR* polymerase chain reaction, *BP* blood pressure, *CTA* CT angiography, *ESR* erythrocyte sedimentation rate, *CRP* C-reactive protein, *DM* diabetes mellitus, *MRA* magnetic resonance angiography, *MVP* mitral valve prolapse

#### Neuropsychiatric and cognitive conditions

Microbial pathogens including *Treponema pallidum*, *Toxoplasma gondii* and group A *Streptococcus* species have long been associated with psychiatric illness although exact pathogenesis is debated [151]. Illnesses with cognitive or neuropsychiatric changes typically occur over a lengthy temporal period, a feature that may relate to chronic infection, through host-immune directed recurrent or persistent activity or by direct pathogen effects on brain function [151]. Similarities exist between neurobartonellosis and autoimmune encephalitis [112]. Conspicuous neuropsychiatric manifestations, including psychosis, aggression, mutism, memory loss, movement disorders and cognitive decline, in the absence of other etiological agents, are common to both conditions. Although few cases are represented in the literature, neurobartonellosis is likely underestimated because of the wide range of clinical presentations possible [4]. Table 10 summarizes cases in which cognitive or neuropsychiatric signs were the primary presentation. Prior history of a cat bite was documented in one case, a 53-year-old man who was treated with a 10-day course of doxycycline for the bite wound before developing confusion, expressive dysphasia and diminished cognitive function a few days after antibiotic completion [152]. These symptoms resolved when treatment was changed to a combination of doxycycline and rifampin, with complete resolution of neurocognitive symptoms by the 8th day of antimicrobial therapy. Two patients presented with sudden onset psychotic behavior and rage. The first, a 14-year-old boy, was diagnosed with pediatric acute onset neuropsychiatric syndrome (PANS) secondary to *Bartonella* infection after months of neuropsychiatric drugs for schizophrenia and treatment for autoimmune encephalitis [153]. *Bartonella* had not been considered until the development of epidermal striae-like lesions, consistent with *Bartonella*-associated cutaneous lesions (BACL) [154]. This patient had serological and molecular evidence of both *B. henselae* and *B. vinsonii* subsp. *berkhoffii* and regained normal function following combination antimicrobial therapy (Table 10). The other patient experienced acute onset of rage, insomnia and personality changes following reported tick bites [155]. The patient was seroreactive to *B. henselae*, although antibiotic therapy was not instituted until further psychiatric illnesses, including panic attacks and major depression, were diagnosed, and the patient demonstrated poor clinical improvement on various psychotropic medications. Interestingly, this patient suffered worsening anxiety following the commencement of antimicrobial therapy but improved substantially over 8 weeks with an adjusted dose of anti-psychotic medication (Table 10). In cases of spirochetal infections, including Lyme borreliosis and syphilis (*T*. *pallidum*), patients often report worsening of clinical signs following administration of antimicrobial therapy, termed Jarisch-Herxheimer reaction [156]. Although this clinical syndrome has not been established in patients suffering from *Bartonella* infections, it has been documented following treatment with doxycycline in other cases and may be worth considering in cases of neurobartonellosis [156].

**Table 10:** Neurobartonellosis cases with primarily neuropsychiatric presentations

CNS neuropathies: neuropsychiatric presentations secondary to *Bartonella* infection							
Age/ sex	Pertinent medical History	Neurological and psychiatric symptoms	Pertinent diagnostics	*Bartonella* serology/PCR	Treatment/ duration	Outcome	Ref.
53/M	Cat bite followed by fever and left inguinal lymphadenopathy	Confusion, expressive dysphasia with diminished speech fluency, recall, word repetition, difficulty with writing and reading comprehension_1_	No initial diagnostics following cat bite	No initial titers	Doxycycline × 10d	Neurological symptoms 3d later	[152]
			Mild ↑WBC (neutrophils) and CRP, ↑CSF protein_2_ Inguinal CT: heterogeneous mass_2_	*Bh* IgG 1:2048 IgM 1:160	Doxycycline + rifampin × 14d	Complete recovery after 8 days of therapy	
14/M	Previous good health	Sudden onset psychotic behavior; hallucinations, delusions, suicidal and homicidal ideation	Extensive_3_	No initial titers	Extensive _4_	No significant improvement	[153]
	Development of skin striae suspected BACL		Skin striae histopathology: lymphohistiocytic infiltration	No pre-antibiotic titers	Doxycycline × 2 months	Continued *Bh* PCR amplification	
				*Bvb* II IgG 1:128 *Bh* PCR + (blood, serum, culture-enriched blood) Sequencing + _5_	+ azithromycin, hydroxychloro-quine, nystatin + rifampin	Gradual reversal of symptoms, withdrawal of anti-psychotic drugs. Repeat *Bh* testing neg	
41/M	Fever and axillary LN pain. Tick attachment	Sudden onset eccentric rage, irritability and insomnia	CDC 2- tier testing Lyme screening neg	*Bh* IgM 1:256	None documented	Worsening signs over 2 weeks	[155]
		Panic attacks, major depression and severe agitation		No additional testing	Valproic acid × 3w, then lithium carbonate × 3w	No improvement	
					Quetiapine	Improvement with ↑ dosage	
					Azithromycin + Rifampin × 8w	Worsening anxiety and panic attacks, controlled by ↑dose of quetiapine; 90% resolution after 8w on antibiotics	

^1^Comprehension of spoken language was intact. Deficits in cognitive function including orientation and registration. More significant deficits in attention and calculation, recall and language

^2^Brain CT and MRI NSF. HSV PCR neg. (CSF). Normal serum Igs. HIV neg. No valvular lesions on TEE. Inguinal mass consistent with enlarged LN. Aspiration revealed purulent exudate, culture and gram stain neg

^3^Autoimmune panels, ESR, CRP, voltage-gated potassium channel antibody, EEG, brain MRI, CSF, antistreptolysinn O, anti-DNase B, *Mycoplasma pneumoniae* IgG and IgM, Lyme ELISA, WB, viral titers

^4^In- and outpatient hospital care, anti-psychotic, mood stabilizing, antidepressant medications, benzodiazepines *Bh*,

^5^*Bvb* I and III, *Bk* and *Bq* titers neg. *Anaplasma*, *Babesia*, *Borrelia burgdorferi* sensu lato, *Ehrlichia*, hemotropic *Mycoplasma* PCR neg

Sixteen s–23 s ITS and rpoB gene target 99.3–100% similar to *Bh* strain Houston 1 (GenBank)

*WBC* white blood cells, *CRP* C-reactive protein, *CSF* cerebrospinal fluid, *CT* computed tomography, *Bh* *Bartonella henselae*, *BACL* *Bartonella*-associated cutaneous lesions, *Bvb II* *Bartonella vinsonii* subspecies *berkhoffii* type II, *PCR* polymerase chain reaction, *LN* lymph node, *CDC* Centers for Disease Control and Prevention

Two recent publications examined patients with psychoses for evidence of *Bartonella* infection. Lashnits et al. evaluated patients with schizophrenia or schizoaffective disorder for the presence of *Bartonella* in blood and found a higher percentage of patients tested positive for the presence of *Bartonella* spp. DNA by droplet digital PCR (ddPCR) (11/17) than healthy controls (1/12) [157]. A study by Delaney et al. (2024) evaluated the association between *Bartonella* species and adult psychosis [158]. A total of 116 patients and controls were evaluated for evidence of *Bartonella* spp. DNA using quantitative PCR (qPCR), digital PCR (dPCR) and ddPCR. Similar to the prior study, there was a higher proportion of adults with psychosis demonstrating evidence of *Bartonella* spp. DNA in their blood (43.2%) compared to non-psychotic adults (14.3%). The species of *Bartonella* was determined for just over half of the bacteremic patients (18/31), and coinfection with different *Bartonella* spp. was also demonstrated in three patients with psychoses. *Bartonella* species represented included *B. henselae*, *B. vinsonii* subsp. *berkhoffii*, *B. quintana*, *B. rochalimae* and *B. alsatica* [158]. The latter two species had not previously been identified in people suffering from neurological conditions. *Bartonella rochalimae* was first diagnosed in a febrile patient in 2007 and has since been detected in two other people, the most recent report from a patient suffering from infective endocarditis with a non-clinical infectious embolism [159]. It has also been documented in dogs with endocarditis in the USA and Europe and from fleas [160–162]. The mammalian reservoir species for *B. alsatica* is wild rabbits [163]. It has been documented to date in three patients, two of whom had culture-negative endocarditis, and one patient with generalized lymphadenitis [163]. Identification of these two novel *Bartonella* species in people suffering from psychoses underscores potential undescribed zoonotic or vector-borne risk for these organisms.

Of note, neither study demonstrated significant differences in *Bartonella* seroreactivity between patients with signs of neuropsychiatric disease and control subjects, and serological results commonly do not correlate with results of molecular testing [157, 158]. It is anticipated that *Bartonella* exposure is common, as seroreactivity in absence of disease has been reported [164–166]. IFA sensitivity is considered low, potentially because of antigenic variation in *Bartonella* strains resulting in false-negative serology [167, 168]. Evidence of immunological dysfunction was demonstrated in two *B. henselae*-infected patients with IgG deficiency, a factor that could also impact false-negative serology [169, 170]. The incongruency between the detection of bacterial DNA through targeted PCR and antibody titers continues to confuse diagnosis and frustrate attempts at formulating a standardized diagnostic protocol, placing the burden on the clinician or the patient to pursue a diagnosis of this stealth pathogen [169]. As the recent manuscripts on neuropsychiatric illness demonstrate, PCR detection of the organism may be the preferred diagnostic test in cases of neurobartonellosis [157, 158].

### Pathogenesis

The neuropathogenic mechanisms related to *Bartonella* infection can be broadly classified as direct, through hematogeneous spread and vascular endothelial cell invasion, and indirect, secondary to its repertoire of immune evasion tactics [171, 172]. In mammals, *Bartonella* infection is characterized by persistent intraerythrocytic infection, but it has shown in vitro capabilities of infecting a range of other cell types, including CD34 + bone marrow progenitor cells, pericytes, microglia, macrophages and dendritic cells. [172–179]. The ability to inhabit vascular endothelial cells may account for persistence through recurrent bacterial seeding and is also anticipated to be a primary pathway for *Bartonella* to enter the central nervous system [63]. Additionally, evidence of the pathogen’s ability to survive in mesenchymal stromal cells, which are involved in vascular angiogenesis, provides a potential further indication of a cellular niche [179]. In vitro studies have shown the ability of *B. henselae* to invade human brain vascular pericytes, which exist embedded in the capillary basement membrane with direct endothelial cell contact, and diminish their proliferation [174]. This could impact vascular permeability through diminished vessel coverage [174, 180]. In fact, CNS diseases including Alzheimer’s disease, amyotrophic lateral sclerosis and stroke are associated with loss or damage of pericytes, and evaluation of infectious mechanisms of pericyte damage should be considered [180–182]. Infection of microglial cells has been documented in vitro in feline cell culture, where viable, intracellular *B. henselae* was cultured up to 28 days post-infection [175]. Other in vitro research using peripheral macrophages as an infection model suggests that macrophages may serve as shuttles (a Trojan horse) for brain entrance [176].

*Bartonella* infection is well known to stimulate production of VEGF, a potent stimulating agent for angiogenesis [96–99]. VEGF would be anticipated to have a protective role in neurocognitive conditions, as it has been shown to diminish capillary loss and promote neurogenesis [183]. Interestingly, brain vascular alterations are pivotal in a variety of neurological conditions aside from stroke, including Alzheimer’s disease, depression and schizophrenia [184–187]. Some studies have found that VEGF serum levels change during the course of the disease. Although the literature depicts some conflicting associations of VEGF on the progression of neurocognitive disease, it is possible that *Bartonella*-associated VEGF-driven vasculoproliferation leads to abnormal vessel structure, which could compound cognitive disorders or impact cellular signaling [184–190]. Interleukin 8, a cytokine that has both angiogenic and chemotactic functions, was also demonstrated to be elevated in human microvascular endothelial cells in vitro within 6 h of *Bartonella* infection, providing further support for the ability of this organism to significantly impact vasculogenesis [191]. Use of VEGF as a biomarker in neurological and neurocognitive disease may be of potential clinical use, and further studies to determine *Bartonella*’s effect on the VEGF family of chemokines would be beneficial in delineating the role of this pathogen in neurocognitive dysfunction [189, 190].

In averting immune detection, one of the mechanisms that *Bartonella* uses is to subvert cellular transcription factor STAT3 (signal transducer and activator of transcription) to enhance production of the canonical anti-inflammatory cytokine, interleukan-10 (IL-10). This cytokine is naturally produced during infection and inflammation and acts as a safeguard to halt chronic activation of the immune response [171]. IL-10 was demonstrated to have inhibitory effects on microglial phagocytosis of amyloid-beta protein in a mouse model of Alzheimer’s disease, with reversal of phagocytosis inhibition later demonstrated in an IL-10 knock-out mouse model [192]. IL-10 from CD4 + T cells has recently been shown to promote CNS inflammation by sustaining survival of effector T cells [193]. In cases of chronic IL-10 overproduction, it may serve to propagate neuroinflammatory changes and inhibit appropriate immune responses, which could enhance neurological damage [194].

Although in most cases of BAND tissue biopsies are not obtained, patients with ischemic stroke secondary to CSD have cerebral arteritis pathology consistent with an immune-mediated process [144, 195]. The modified lipopolysaccharide component of *Bartonella*’s outer membrane is poorly recognized by Toll-like receptor 4, an innate immune receptor involved in pathogen clearance [171]. Additionally, bacterial virulence factors, including BadA (*B. henselae*) and the Vomp outer membrane proteins (*B. quintana*), avoid host immunity through antigenic variation [196]. It is possible that immune-mediated attacks on host cells could arise because of molecular mimicry secondary to *Bartonella*’s sub-inflammatory cellular components [197]. Bystander activation, which describes activation of local inflammatory cells leading to tissue injury in the presence of an infection, may be another mechanism by which *Bartonella* imparts neurological tissue damage [197].

### Neuropathology

Due to the development of less invasive diagnostic techniques, biopsies of the central nervous system have been rarely performed in neurological patients infected with *Bartonella* spp. Histological lesions have been described in the meninges and/or different regions of the brain. Granulomatous meningoencephalitis with prominent perivascular lymphocytic infiltrates has been noted in the right thalamus of a 19-year-old man [129]. There was no evidence of bacteria with a Warthin-Starry stain, which is a silver stain used in the detection of *Bartonella*, and spirochetes including *Borrelia*. Nevertheless, infection with *B*. *quintana* was identified by PCR. Notably, mild gliosis was the only finding initially reported with a stereotactic biopsy. Two fatal cases of disseminated *B*. *henselae* infection with encephalitis have been published: both concerned children with initial lymphadenitis, the typical symptom of CSD [114, 198]. At the microscopic evaluation of necropsy samples, lesions were observed in the lymph nodes, spleen and brain; lung, liver and meninges were additional lesions reported in case 1. Anatomic location of the brain lesion was specified only for case 2: the frontal, parietal and occipital lobes and the pons contained lesions, characterized by perivascular lymphocytic infiltrates (cases 1 and 2) and glial nodules (case 2). Molecular evidence of *B*. *henselae* and visualization of the bacteria with a Warthin-Starry stain were successful in the brain for only case 1. Cerebral vasculitis with secondary infarction has been rarely reported in *Bartonella*-infected patients [144, 195]. A vascular lesion involved the right parietal lobe in an 11-year-old girl. For this case, *Bartonella* was visualized with confocal laser scanning microscopy, and there was amplification of *B*. *henselae* DNA from the formalin-fixed paraffin-embedded brain biopsy. Perivascular infiltrates, mainly composed of T lymphocytes and plasma cells, were also seen at the periphery of the lesion. Focally extensive granulomatous meningitis, with multinucleated giant cells, has been described in two adults infected with either *B*. *henselae* or *B*. *quintana* [199, 200]. The granulomatous meningitis lesion led to secondary brain atrophy by compression. No bacteria were identified with Gram staining in direct smears, but PCR was positive in both cases. One patient was frequently scratched by his cat on the scalp, suggesting direct extension as potential portal of entry for *Bartonella*.

### Diagnostic considerations

For diagnostic confirmation of neurobartonelloses, it is generally accepted that the higher sensitivity of molecular diagnostic methods, such as qPCR and digital PCR, are preferred to, or are used in addition to, other conventional direct detection methods such as culture isolation, biochemical identification and microscopic visualization [201–204]. Current molecular methods for the detection of *Bartonella* spp., like assays for many other vector-borne pathogens, are usually laboratory-developed tests rather than commercially available and federally approved diagnostic test kits, most often used in commercial diagnostic laboratories. As such, establishing ideal diagnostic specimens for testing, variability in pathogen DNA amplification in various patient samples (blood, cerebrospinal fluid, pathological effusions, tissues), DNA stability in diagnostic specimens during shipment or storage, standardization of PCR protocols, assessment of laboratory contamination risks and molecular assay sensitivity compared to conventional assays (including cost-effectiveness and availability) are among the biggest challenges for a particular molecular detection method to become the preferred or reference diagnostic method. Despite these and other limitations, molecular-based assays have played an increasingly significant role in our evolving understanding of neurobartonelloses. As documented by a number of research laboratories, combining a sample enrichment step with PCR can further improve the sensitivity of detecting *Bartonella* spp. DNA in a patient specimen [36]. Although there are minimal data, enrichment culture of CSF has facilitated the diagnosis of neurobartonelloses and has in some instances facilitated bacterial isolation from the patient’s CSF [153]. Clearly, additional research is needed to improve the molecular (pathogen DNA-based) diagnosis of neurobartonelloses, which is challenging because of the numerous and genetically diverse *Bartonella* spp.

Due to its historical acceptance and relative simplicity, serology has been used most often for the diagnosis of neurobartonelloses [202, 204]. Due to limitations associated with serology, results must be interpreted carefully in the diagnostic setting, and whenever possible the diagnosis should be further supported by one or more direct detection methods [203, 204]. Selected limitations include differences in immune response (antibodies may not be detectable in the early stages of the disease, and some infected patients do not have detectable antibodies despite being chronically infected), selection of the appropriate antigen (both at *Bartonella* species and strain levels), variability in pathogen antigen expression over time in the patient and lack of standardization between laboratory protocols (i.e. cut-off values). Antigen preparation for indirect immunofluorescent assays requires isolates (considering > 50 *Bartonella* spp.), is labor intensive and time-consuming and requires specially trained personnel working in appropriate biocontainment settings.

### Treatment considerations

Standardized treatment protocols have not yet been established for the various neurobartonellosis disease presentations [203, 204]. In addition, in vitro antibiotic susceptibility testing has only been performed on a small number of the *Bartonella* spp. that infect humans, predominantly *B. bacilliformis, B. henselae* and *B. quintana* [205–211]. The extent to which in vitro susceptibility data correlate with treatment efficacy in patients deserves future research consideration. A manuscript by Zheng et al. (2020) evaluated the in vitro efficacy of a variety of antimicrobial drugs, as single agents and in combination, against *B. henselae* growing in a stationary phase and in biofilm [206]. Combinatory antimicrobials, including azithromycin/ciprofloxacin and rifampin/ciprofloxacin, were found to kill stationary phase bacteria after 24 h of exposure and to eradicate *B. henselae* biofilm after 6 days of treatment, which may explain the response to multi-drug therapy in patients suffering from chronic neurobartonelloses [206]. Due to the increasingly large number of *Bartonella* spp., the considerable number and diversity of animal reservoir hosts, and documented or suspected transmission by several arthropod vectors, people are more frequently exposed to this genus of bacteria than has been historically appreciated [1, 2, 178]. In most instances, host immunity eliminates *Bartonella* prior to or during an acute infection, such as cat scratch fever [208]. Therefore, antibiotics are not routinely administered for uncomplicated cat scratch fever or *Bartonella*-associated neuroretinitis. However, bloodstream infection in blood donors from Brazil supports the ability of *B. henselae* to cause persistent intravascular infection in healthy individuals [166]. In patients, isolation or repeated documentation of *Bartonella* spp. DNA in blood, CSF, synovial fluids, pathological effusions or tissues supports a role for these bacteria in chronic infections, a concept that is not universally accepted by many practicing physicians. Based upon case reports, therapeutic elimination of *Bartonella* spp. from the blood or nervous tissues of some patients with neurological symptoms is more difficult to achieve than is generally appreciated [212]. Patients with neurological symptoms have failed to eliminate the bacteria from blood following several weeks of combination antibiotic therapy and after 6 months of doxycycline (single antibiotic) treatment [212]. Based upon currently available testing modalities, pre-treatment diagnostic confirmation of neurobartonellosis via culture or molecular confirmation of the presence of pathogen RNA or DNA is challenging; therefore, proving therapeutic elimination of the bacteria is technically more difficult to achieve, emphasizing the importance of long-term patient follow-up in the clinical setting.

### Prevention

Currently, no vaccines are available for the prevention of infections with individual or multiple *Bartonella* species. Researchers are investigating potential vaccine targets for *B. bacilliformis, B. henselae* and *B. quintana* [213–215]. Whether, or the extent to which, effective vaccines can be developed, or will be utilized if developed, will ultimately be determined by improved understanding of the medical importance of this genus of bacteria. Most importantly, we need to understand the extent to which these bacteria contribute to chronic, insidious or relapsing illnesses, including involvement of the cardiovascular, musculoskeletal and nervous systems. As infection with the same *Bartonella* species has been reported in multiple family members, it is important to investigate other modes of transmission, including blood transfusion, sexual, transplacental and salivary [38]. As discussed previously, arthropod vector and animal exposures are definite risk factors for acquiring neurobartonelloses. Therefore, as reviewed in depth elsewhere, avoiding arthropod bites and animal bites and scratches are important prevention strategies [1, 2, 178, 216].

## Conclusions

Although neurobartonelloses are emerging from obscurity, there remains a substantial need for research that addresses the neuropathogenesis, optimal diagnostic approaches, defined treatment regimens for various neurological presentations and prevention strategies. As chronic bacteremia has been confirmed with several *Bartonella* spp. in patients and healthy individuals, it is critical that physicians and researchers investigate and define the role of these bacteria not only in association with acute disease presentations but also in patients with chronic, incompletely understood neurological and neuropsychiatric illnesses.

## Data Availability

No datasets were generated or analysed during the current study.
